# Preference and strategy in proposer’s prosocial giving in the ultimatum game

**DOI:** 10.1371/journal.pone.0193877

**Published:** 2018-03-05

**Authors:** Misato Inaba, Yumi Inoue, Satoshi Akutsu, Nobuyuki Takahashi, Toshio Yamagishi

**Affiliations:** 1 Center for Experimental Economics, Kansai University, Suita, Osaka, Japan; 2 Faculty of Economics, Teikyo University, Hachioji, Tokyo, Japan; 3 Graduate School of International Corporate Strategy, Hitotsubashi University, Chiyoda-ku, Tokyo, Japan; 4 Graduate School of Letters, Hokkaido University, Sapporo, Hokkaido, Japan; Groupe ESC Dijon Bourgogne, FRANCE

## Abstract

The accumulation of findings that most responders in the ultimatum game reject unfair offers provides evidence that humans are driven by social preferences such as preferences for fairness and prosociality. On the other hand, if and how the proposer’s behavior is affected by social preferences remains unelucidated. We addressed this question for the first time by manipulating the knowledge that the proposer had about the responder’s belief concerning the intentionality of the proposer. In a new game called the “ultimatum game with ambiguous intentions of the proposer (UG_AMB_),” we made the intentionality of the proposer ambiguous to the recipient. We expected and found that the proposer would make more unfair offers in this new game than in the standard ultimatum game. This expectation can be derived from either the preference-based model or the strategy model of the proposer’s giving decision. The additional finding that more unfair giving in the UG_AMB_ was not mediated by the proposer’s expectation that the recipient would be more willing to accept unfair offers provided support for the preference-based model. Using a psychological measure of cognitive control, the preference-based model received additional support through a conceptual replication of the previous finding that cognitive control of intuitive drive for prosociality in the dictator game, rather than mind reading in the ultimatum game, is responsible for the difference in giving between the two games.

## Introduction

It is widely shared by social and biological scientists that humans are a highly cooperative species [1–3]. This understanding is based on accumulation of evidence that human behavior is driven not only by self-interest, but also by social preferences such as concerns for fairness and others’ welfare [4–6]. One area where the critical role of social preference has been investigated is the study of economic games called the dictator game (DG) and the ultimatum game (UG). The DG is played by two players—a “dictator” and a “recipient.” The dictator is endowed with some money, and is asked to share it with the recipient in any way he/she prefers. Usually, the game is anonymously played once between two players, such that the dictator does not need to think about the possibility that his/her future welfare may be affected by the recipient’s responses. In this sense, it is a game of pure preference; that is, only the dictator’s preference for the reward distribution affects his/her choice. The non-zero giving to the recipient provides evidence that the dictator cares about the recipient’s welfare.

The UG is like the DG with one key difference. It is also played by two players: a “proposer” and a “responder.” The proposer decides how much of an endowment to offer to the responder. The responder has two options: to accept the proposer’s offer or to reject it. If the responder accepts the proposer’s decision, each earns according to the proposer’s decision. If the responder rejects the decision, neither earns any money. The responder who is totally driven by self-interest should accept any non-zero share of the endowment. However, a proposer’s offer of 30% or less of an endowment to the responder is often rejected in most Western societies [7, 8]. This finding is typically considered evidence that humans are at least partly driven by some social preference such as preference for fairness [9–11]. Furthermore, the finding that the responder seldom rejects unfair offers when they do not reflect malignant intent of the proposer [9, 10, 12–15] provides additional evidence that the responder’s rejection of an unfair offer in the UG is driven by social preferences.

While the influence of social preferences in the responder’s behavior has been well documented [4, 12, 15, 16], how they influence the proposer’s behavior has hardly been reported. In this study, we focused on this overlooked proposer’s behavior in the UG. Although the UG is a game of pure preference for the responder, it is not necessarily the case for the proposer. Even the proposer who is entirely dictated by self-interest will not necessarily take most of the endowment. This is because the anticipation that the responder may reject an unfair offer provides an incentive for the proposer to propose an offer that is acceptable to the recipient. In this sense, the UG for the proposer is a strategic game that involves reading the responder’s mind and adjusting his/her behavior to the responder’s anticipated response. The difference of giving in the DG and the UG is thus attributed to the strategic considerations of the proposer and the lack thereof of the dictator [17–19]. Previous studies have shown significant differences in giving between the two games, thus providing support for a strategy model of the proposer’s behavior in the UG [17, 18]. Additional support for strategic considerations comes from the finding that young children, who do not anticipate rejection of unfair offers by the recipient, do not provide a fair share in the UG [20, 21].

The first purpose of this study is to examine how proposers’ social preferences and their strategic considerations combine in their decision making. The level of giving is known to be higher in the UG than in the DG [17–19], and this difference is typically attributed to strategic considerations in the UG and the lack thereof in the DG. However, this difference may also reflect the differential activation of prosocial preferences in the two games. According to the intuitive prosociality model of human cooperation [22–25], prosocial choices are made intuitively, while selfish choices are made when the intuitive drive for prosocial choices is cognitively suppressed through deliberative scrutiny of the immediate incentives of the situation. Earlier studies [26, 27] that compared giving in the DG and UG in terms of the cortical thickness of the dorsolateral prefrontal cortex (DLPFC), a region of the brain that is considered responsible for cognitive control, provided support for the suppression of intuitive prosocial drive account of the DG–UG difference in giving.

We use the presence and absence of malignant intention in the proposer’s offer to assess the influence of social preferences in the proposer’s decision. The effect of the proposer’s non-malignant intentionality on the responder’s rejection behavior has been tested in earlier studies by comparing rejection rates in the standard UG (UG_STD)_ and a variant of the UG, in which all proposers simply relayed the decisions made by the computer (UG_NINT_) [9, 28]. However, in this comparison, it is not possible to test the influence on the ***proposer***’s giving because the proposer does not make any decision in the UG_NINT_. Facing this difficulty, we designed a new variant of the UG, in which the proposer knows that the responder is not certain about the intention behind the proposer’s unfair offer. We call this game the UG with ambiguous proposer’s intention (UG_AMB_). Specifically, responders in the UG_AMB_ were told that some proposers intentionally made the offer, while others were simply relaying the offer that was made by the computer. Therefore, the responder in the UG_AMB_ who faced an unfair offer was not certain if the offer had been intentionally made by the proposer or not. How the responder was informed about the intentionality of the proposer’s offer was also mentioned to the proposer.

The use of the UG_AMB_ allows us to examine the effect of non-malignant intentionality on the proposer’s giving, which is not possible with the use of the UG_NINT_. Proposers in the UG_AMB_ make their own offers with full understanding that the recipient is not certain if the proposer’s offer is intentionally made or not. We compared the proposer’s decisions in the UG_AMB_ with those in the UG_STD_, in which it is common knowledge to both the proposer and recipient that the distribution of endowment is intentionally made by the proposer. Given the standard finding that responders tend not to reject unintentionally made unfair offers [9, 10, 13–15], they would also be less inclined to reject unfair offers in the UG_AMB_ compared to in the UG_STD_. Before testing the effect of ambiguous intentionality on the ***proposer***’s giving, we first examined, as a manipulation check, if the intentionality manipulation affected the ***recipient***’s responses—that is, the level of the minimum acceptable offer (MAO) in the UG_AMB_ (lower willingness to reject unfair offers: “I don’t want to reject even small offers because my partner–proposer may not be responsible for it”) compared to that in the UG_STD_. We then examined if the proposers who anticipated lower probability of rejection by the responder in the UG_AMB_ would lower their levels of offer to the recipient.

The second purpose of this study was to examine if intuitively driven choices—rejection of unfair offers by the respondent and prosocial giving by the proposer—are more strongly suppressed by those with stronger cognitive control ability. According to the intuitive prosociality model of prosocial behavior [22–25], humans are evolutionarily endowed with a behavioral tendency to cooperate in social exchanges by default, and choose not to cooperate only after scrutinizing incentives surrounding the social exchange and finding out that not cooperating produces no negative long-term effect on their future welfare (such as the one-shot nature of social encounters). Therefore, according to the intuitive prosociality model, how strongly the proposer’s giving behavior is suppressed in the DG compared to the UG should reflect his/her cognitive control ability, namely, the ability to suppress intuitive cooperation and pursue self-interest in a situation such as an anonymously played one-shot DG, in which incentives for the players are clearly delineated.

To test the modulating role of cognitive control ability, we measured our participants’ levels of cognitive control with the cognitive reflection test (CRT). The CRT has been proposed as a measure of individual differences in the strength of the function of System Two in the dual process model [29–31]. It is designed to measure people’s ability to refrain from intuitive response in a reasoning context. Earlier studies have demonstrated that the CRT score correlates with other measures of rational thinking [32–34]. Positive correlations that were found between acceptance of unfair offers in the UG_STD_ and the CRT are considered evidence that the CRT measures the ability to override intuitive drives for rejecting unfair offers [16, 35]. Similarly, the CRT score was found to negatively correlate with giving in the DG, and this negative correlation was interpreted to show the effect of cognitive control on suppressing intuitive drive for prosociality [36]. The CRT was also found to play the impulsivity prohibition role that is processed in the DLPFC [37]. It has been reported that the CRT score decreases when the activity of DLPFC is inhibited using transcranial direct currency stimulation [37].

**Fig 1:**
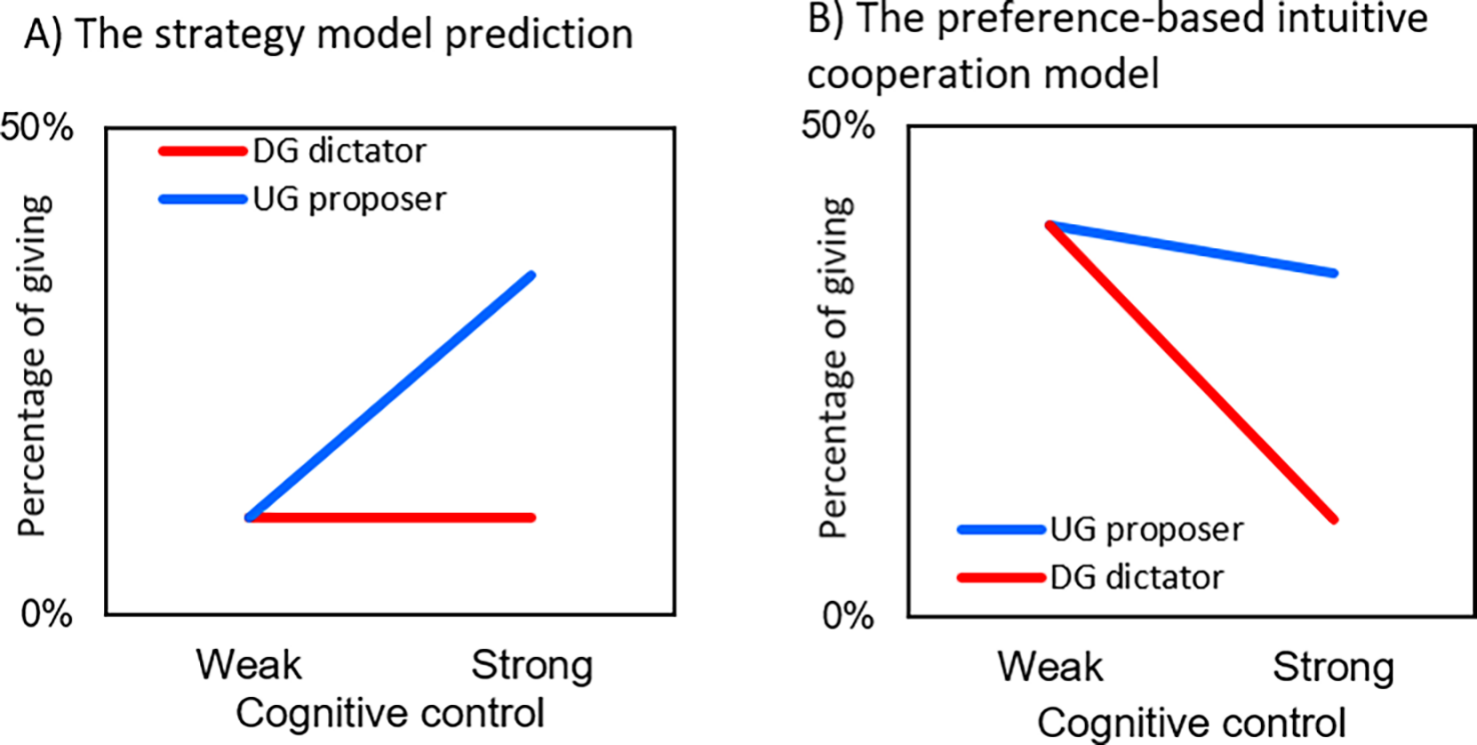
Graphical representations of the two models of the dictator’s giving in the dictator game and the proposer’s giving in the ultimatum game as a function of their level of cognitive control.

If the proposer is a strategic pursuer of self-interest, the proposer with strong cognitive control ability should give a share of the endowment acceptable to the responder; however, the proposer who is lacking cognitive control ability fails to suppress the intuitive drive for self-interest and gives a blatantly unfair offer to the responder [11, 37, 38]. Fig 1A graphically presents this strategy model prediction. The difference in giving between the two games should be the largest among those who are strong in cognitive control, and the heightened difference is expected to relate to increased giving in the UG_STD_ compared to giving in the DG among those with strong cognitive control ability.

Fig 1B presents the prediction based on the alternative, preference-based intuitive cooperation model [22–26]. According to this model, both the DG dictator and the UG proposer behave according to the prosocial preferences when their decisions are not cognitively intervened. Strong cognitive intervention mobilized to secure self-interest suppresses giving in the DG, because giving nothing is clearly the best way to secure self-interest in the one-shot DG. At the same time, proposers who are capable of cognitively controlling their prosocial preferences in the UG give a minimum share of the endowment that they think is acceptable to the responder. A study by Yamagishi and colleagues [26] first replicated the earlier finding by Steinbeis and colleagues [18] that the difference in giving between the two games is larger among those with thicker than thinner DLPFC (which is common to both models, Fig 1A and Fig 1B). Then, they showed that the increasing difference in giving between the two games was produced mostly by a rapid decrease in giving in the DG (Fig 1B), rather than the increase in the UG (Fig 1A), among those with the thicker DLPFC. Comparable results were also reported in other studies [27, 39]. The second purpose of our study is to critically test the two predictions depicted in Fig 1A and 1B using the CRT as an alternative measure of cognitive control. Specifically, we will examine if the CRT score negatively correlates with giving in the DG, but not in the UG. We will further investigate the modulating role of CRT in the effect of intention manipulation on giving and the willingness to reject unfair offers.

## Methods

### Ethics statement

Each participant provided informed, written consent before beginning the experiment. The study was approved by Institutional Review Board of the Center for Experimental Research in Social Sciences at Hokkaido University (No. H28-3) and was conducted in accordance with the Declaration of Helsinki.

### Participants

One hundred and twenty-one undergraduate students (55 women) were randomly selected from a participant pool at Hokkaido University. Participants played four economic games—the DG and three variations of the UG—each time with a newly matched partner. After all games were over, each participant was randomly matched with another participant in each of the two games, and was paid for their earnings in each game played with a randomly matched partner, in addition to a show-up fee of JPY 500 (1 USD ≈ JPY 115).

### Experimental procedure

Participants (6 to 12 in a session) were seated in a classroom, each surrounded by a Π-shaped partition, which prevented them from seeing each other. They played the four games in the same order: the DG, followed by the UG_STD_, the UG_NINT_, and the UG_AMB_. Each participant was provided with a tablet computer to use in the experimental games. Instructions for each game were displayed on the front screen as a series of PowerPoint presentations and were read aloud by an experimenter to ensure that participants understood that other participants were receiving the same instructions. During and after the instructions, participants were encouraged to ask questions. Once the experimenter had ensured that everyone understood the game, participants were asked to enter their decisions on the tablet computer. When all participants finished entering their decision, the experimenter started the instructions for the next game. Participants did not receive any feedback until all four games were over and they finished answering the post-experimental questionnaire. This procedure ensured that all decisions were made without being influenced by other participants’ choices in the previous games. Each game involved two roles. Each participant was paid his or her earnings in randomly selected two of the four games, one as a proposer and the other as a recipient/responder, according to the following schema. First, each participant formed a pair with another in each of the four games, his/her payments in each game as a dictator/propose and a recipient/responder were determined, and two of the four games and the participants’ roles in the two games were selected for actual payment. Participants were informed of these procedures in advance. An experimental session took approximately 90 minutes. The English translation of the instructions for the following four games are provided in S1 Table.

#### Game 1: DG

All participants first played a one-shot DG as dictators. Each participant was endowed with JPY 1,000 (about USD 8.50) and decided how much of the endowment to give to the recipient, in increments of JPY 100.

#### Game 2: The standard UG (UG_STD_)

Participants played a one-shot UG with another participant, first as a proposer and then as a responder. First, each participant decided, as a proposer, how much of an endowment of JPY 1,000 to provide to a responder, in increments of JPY 100. Then, each decided, as a responder, on the minimum acceptable share of the endowment.

#### Game 3: UG with no intentional decision by the proposer (UG_NINT_)

Participants played a one-shot UG, first as a proposer and then as a responder. The decision of the proposer was decided by a computerized lottery, not by the proposer’s choice. Each participant was instructed first to “act as a proposer” and to draw a lottery, which determined the offer to make to the recipient. The instruction, however, did not specify the range from which the lottery was to be drawn (e.g., the whole range between JPY 0 and JPY 1,000). In fact, the computerized lottery was programmed to make an unfair offer (either JPY 200 or 300 to the responder, randomly determined with equal probability). Each participant then decided, as a responder, on the minimum acceptable share of the endowment.

#### Game 4: UG with ambiguous intention of the proposer (UG_AMB_)

Finally, participants played a one-shot UG, first as a proposer and then as a responder. As proposers, all participants made decisions themselves. However, they were told that the decisions of some participants would be replaced by decisions made by a lottery, so that the responder would receive either the proposal made by the lottery or the proposal made by themselves. As proposers, participants decided how much of an endowment (JPY 1,000) to offer to the responder (as in the UG_STD_), with an understanding that a half of their offers would be replaced by a randomly determined one (as in the UG_NINT_). As responders, participants decided on the MAO without knowing that the offer they received would be one intentionally made by a matched proposer or one made by a lottery. All participants knew about this when they made decisions as proposers.

#### CRT

The CRT [29] was administered as a part of the post-experimental questionnaire. It comprised three quizzes. One example was: “A bat and ball cost $1.10 in total. The bat costs $1.00 more than the ball. How much does the ball cost?” The correct answer is 5 cents. The intuitively salient answer is 10 cents. Other answers were neither correct nor intuitively salient. We first excluded 5 participants from the analysis of CRT, who did not provide any answer to one or more quizzes. Then, we counted the number of correct answers and intuitively incorrect answers, and calculated the CRT score by subtracting the latter from the former, because some of the incorrect answers are not intuitively appealing. We believe this method is more consistent with the conceptual definition of the CRT that is supposed to measure how strongly intuitive responses are controlled. See S2 Table for the analysis results using the number of the correct answers or the number of intuitively incorrect answers separately.

## Results

### Own MAO and anticipated rejection likelihood of unfair offers in the UG_NINT_ or the UG_AMB_ are lower than those in the UG_STD_

**Fig 2:**
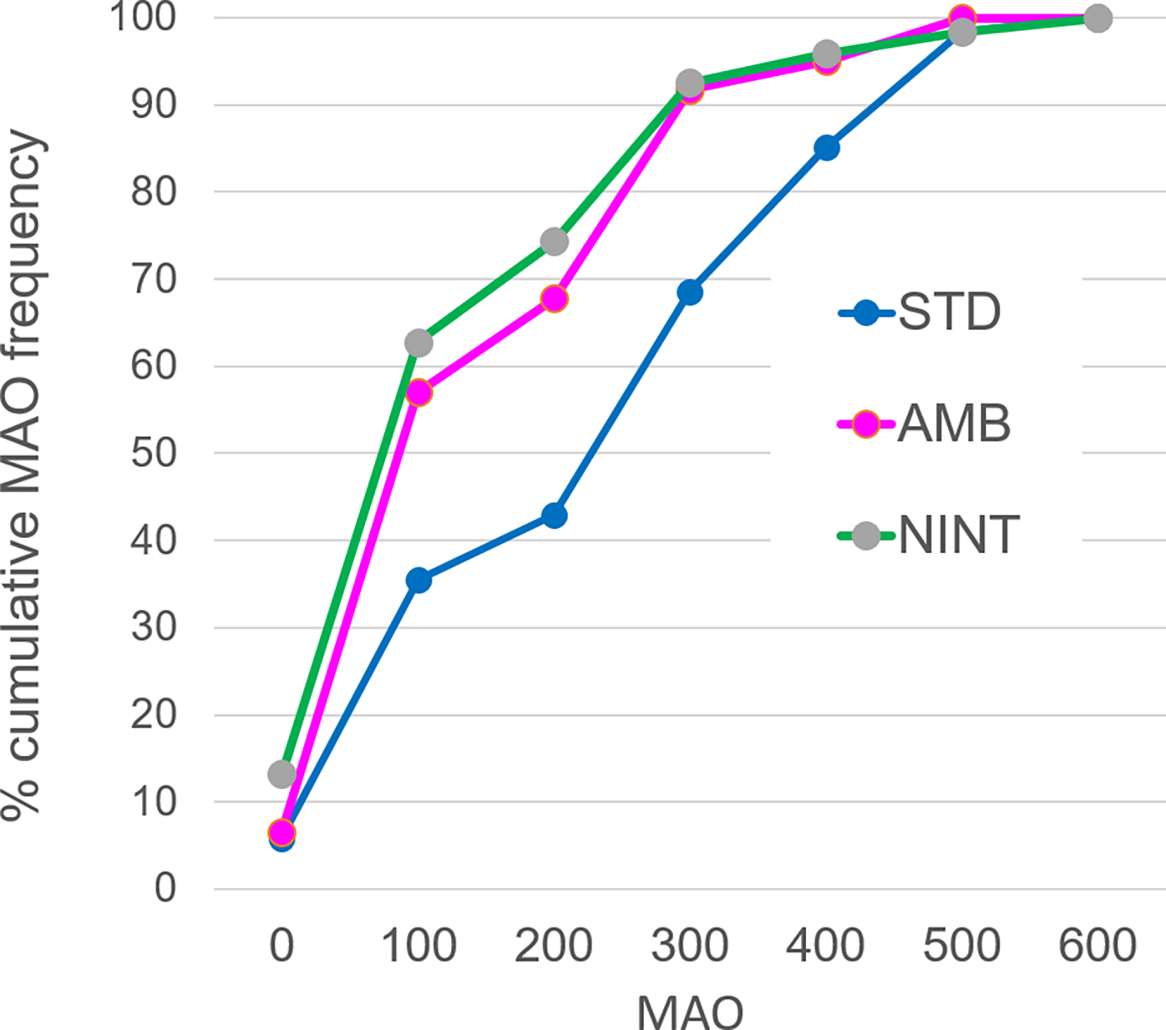
Cumulative distribution of MAO in the UG_STD_, the UG_AMB_ and the UG_NINT_. The vertical axis represents the percentage of responders whose MAO is the JPY shown on the horizontal axis or less.

As a preliminary analysis, we first examined if MAO in the UG_AMB_ was lower than that in the UG_STD_. If not, examining the effect of the ambiguity of intention on the ***proposer***’s offer—our primary focus—would be meaningless. Fig 2 displays the cumulative distribution of participants’ own MAO when they played the role of the responder. The main effect of the three games on the mean MAO (not including DG in which the recipient did not have the rejection option) was highly significant (*F*(2, 240) = 51.71, *p* < .0001, *η*^2^ = .301). MAO in the UG_STD_ was higher than that in the UG_NINT_ (difference *M* = 100.83 ± 24.54, 95% confidence interval), *F*(1,120) = 66.20, *p* < .0001, *η*^2^ = .356) or the UG_AMB_ (difference *M* = 81.82 ± 22.65, *F*(1,120) = 51.16, *p* < .0001, *η*^2^ = .299). The latter difference provides us with a foundation to expect more unfair offers by the proposer in the UG_AMB_ than in the UG_STD_. The difference in the mean MAO between the last two games in which the intention of the proposer was absent (UG_NINT_) or ambiguous (UG_AMB_) was relatively minor, though statistically significant (difference *M* = 19.01 ± 13.81, *F*(1,120) = 7.43, *p* = .007, *η*^2^ = .058). Non-parametric analyses results with post-hoc adjustment of multiple comparisons are shown in S3 Table, confirming the above results of the ANOVAs.

### Giving in the UG_AMB_ is lower than that in the UG_STD_

**Fig 3:**
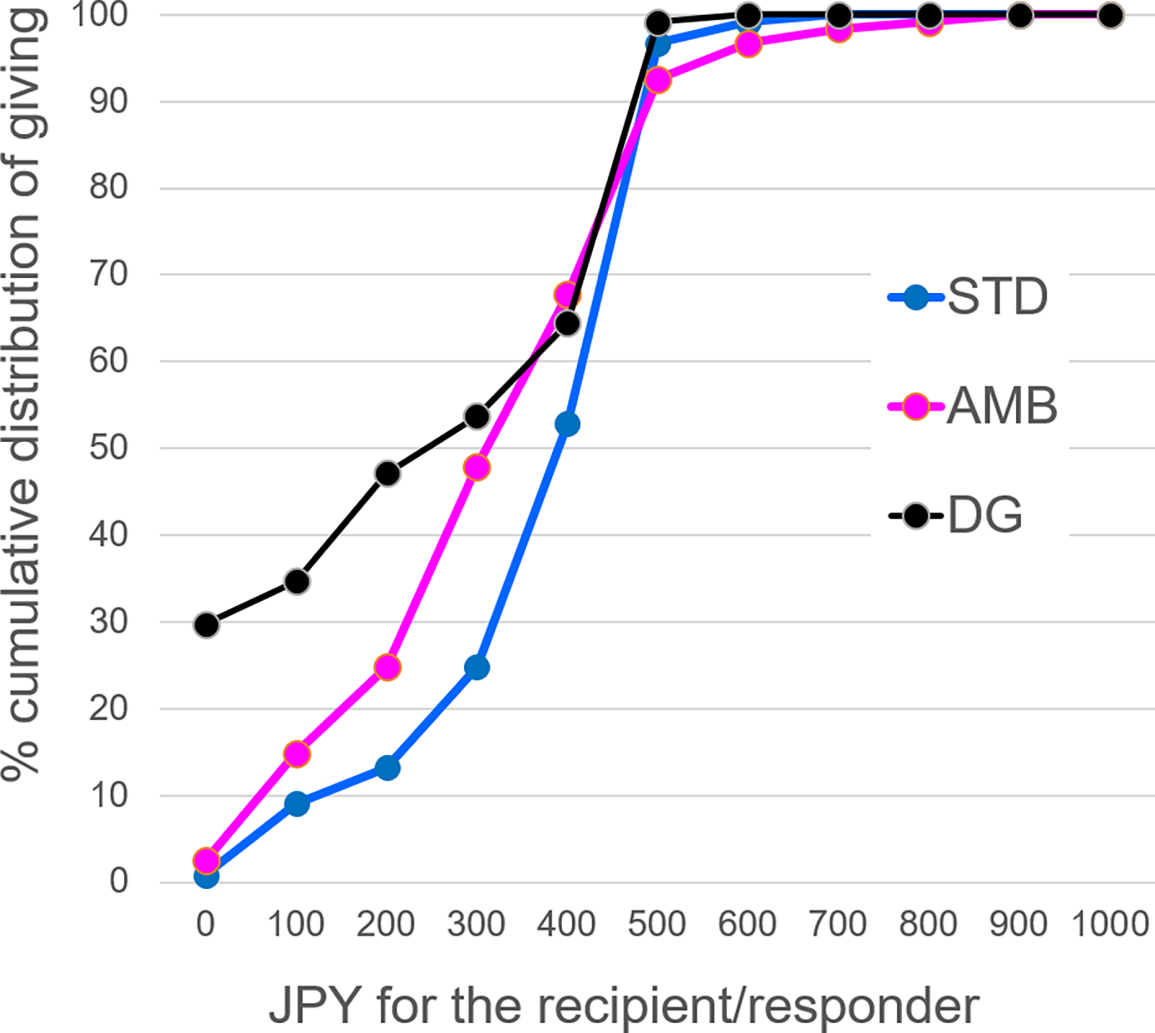
Cumulative distribution of giving in the three games (DG, UG_STD_, and UG_AMB_). The vertical axis represents the percentage of responders whose (proposed) giving to the recipient (responder) is the JPY shown on the horizontal axis or less.

Next, we examined our primary hypothesis that the ambiguity of malignant intention would make the proposer give less to the responder. Fig 3 shows the cumulative distribution of giving in the three games (DG, UG_STD_, and UG_AMB_) excluding UG_NINT_ in which participants did not make the giving decision. The main effect of the game was highly significant (*F*(2, 240) = 31.27, *p* < .0001, *η*^2^ = .207). Giving in the UG_AMB_ fell in-between giving in the DG (difference *M* = 84.30 ± 38.25, *F*(1,120) = 19.04, *p* < .0001, *η*^2^ = .137) and giving in the UG_STD_ (the difference *M* = 47.93 ± 39.89, *F*(1, 120) = 15.72, *p* = .0001, *η*^2^ = .116).

### Own MAO and anticipated rejection of unfair offers do not mediate the effect of ambiguous intention on giving in the UG_AMB_

The correlations between giving, MAO, and anticipated likelihood of others’ rejection of unfair offers in each game and across games are reported in Table 1. The level of giving as a proposer in the UG_STD_ was strongly correlated with own MAO in the same game (*r* = .46, *p* < .0001). That is, those who are intolerant of being treated unfairly behaved in a fair manner as proposers, and vice versa. In addition to MAO, we asked in the post-experimental questionnaire what percentage (in increments of 10%) of the other participants would reject a share of JPY 200 (20% of the total endowment). This measure of the anticipated rejection likelihood of an unfair offer by other players in the UG_STD_ was also significantly correlated with giving in the UG_STD_ (*r* = .38, *p* < .0001). In contrast to the high correlation between own MAO or anticipated rejection likelihood and giving in the UG_STD_, the level of giving in the UG_AMB_ was not significantly correlated with own MAO in the UG_ABM_ (*r* = .17, *p* = .063). Interestingly, however, giving in the UG_AMB_ was more strongly correlated with MAO in the UG_STD_ (*r* = .41, *p* < .0001) rather than in the same UG_AMB_; the difference in the correlation coefficients was significant (*z* = 2.04, *p* = 0.042). Similarly, giving in the UG_AMB_ was more strongly correlated with the anticipated likelihood of rejection in the UG_STD_ (*r* = .30, *p* = .001) than in the UG_AMB_ (*r* = .24, *p* = .007), although the difference was not statistically significant (*z* = 0.44, *p* = 0.660). The stronger relationship of giving in the UG_AMB_ with MAO in the UG_STD_ (sensitivity to intentionally unfair treatment) than with MAO in the same game (UG_AMB_) is inconsistent with the strategic view of lower giving in the UG_AMB_, because the MAO or anticipated rejection likelihood in the same game should be used to make strategic decisions. Furthermore, this finding provides support for the preference-based interpretation, because it suggests that the participant’s sensitivity to unfair treatment which is most effectively captured by the participant’s reaction to unfair treatment in the UG_STD_, in which the proposer’s malignant intention is transparent, plays a more important role in his/her rejection decision in the UG_AMB_.

**Table 1:** Correlations between giving, MAO, and expected rejection probability of unfair offers within and across games. The diagonal entries are the means.

		Giving			MAO			Expected rejection prob.		
		DG	UG_STD_	UG_AMB_	UG_STD_	UG_AMB_	UG_NINT_	UG_STD_	UG_AMB_	UG_NINT_
Giving	DG	271.04	0.385***	0.399***	0.132	-0.045	-0.042	0.120	-0.010	0.007
	UG_STD_	0.385***	403.31	0.637***	0.462***	0.228*	0.132	0.382***	0.308***	0.235**
	UG_AMB_	0.399***	0.637***	355.37	0.411***	0.170	0.124	0.295***	0.243**	0.221*
MAO	UG_STD_	0.132	0.462***	0.411***	263.64	0.630***	0.570***	0.713***	0.563***	0.518***
	UG_AMB_	-0.045	0.228*	0.170	0.630***	181.82	0.821***	0.652***	0.722***	0.695***
	UG_NINT_	-0.042	0.132	0.124	0.570***	0.821***	162.81	0.583***	0.689***	0.715***
Expected rejection probability	UG_STD_	0.120	0.382***	0.295***	0.713***	0.652***	0.583***	45.372	0.714***	0.659***
	UG_AMB_	-0.010	0.308***	0.243**	0.563***	0.722***	0.689***	0.714***	31.157	0.916***
	UG_NINT_	0.007	0.235**	0.221*	0.518***	0.695***	0.715***	0.659***	0.916***	27.686

*p < .05

**p < .01

***p < .001.

Further evidence against the strategic interpretation comes from the absence of a significant correlation between the effect of intention manipulation on giving (giving in the UG_STD_–UG_AMB_) and the same effect on MAO (MAO in the UG_STD_–UG_AMG_) (*r* = -.09, *p* = .343) or on other players’ anticipated willingness to reject unfair offers (*r* = .01, *p* = .913). That is, the effect of the intention manipulation on giving was independent of its effect on MAO or anticipated others’ rejection likelihood, and thus the latter effect cannot mediate the former. These findings suggest that our players did not strategically use own MAO or anticipated rejection likelihood in making offers to their responders. Rather, those who prefer fairness and who were more willing to give in both UG_STD_ and UG_AMB_ were the ones who were more strongly upset by intentionally unfair treatment in the UG_STD_.

This interpretation of the above analysis results, however, requires caution because the expectation measure was not incentivized. That is, participants were not provided with an incentive to carefully assess others’ willingness to reject an unfair offer and report it in the post-experimental questionnaire. While an offer of JPY 200 or less (i.e., the cumulative MAO of JPY 300) was rejected by 57.02% of responders in the UG_STD_, the mean expected probability of rejecting an offer of JPY 200 or less was 45.37%; participants generally underestimated the rejection probability by others in the UG_STD_. On the other hand, 32.23% of responders in the UG_AMB_ and 25.62% in the UG_NINT_ rejected the share of JPY 200 or less expecting on average that 31.16% of other responders in the UG_AMB_ and 29.69% in the UG_NINT_ would reject the offer. The tendency for underestimation was not clear in those games; however, some participants substantially overestimated the probability of rejection in these two games. For example, 10 participants in the UG_AMB_ and 11 participants in the UG_NINT_ expected that 80% or more responders would reject the offer of JPY 200 or less. When these extreme over-estimators were excluded from the analysis, the correlation between giving and MAO (*r* = 0.20, *p* = .042) or expectation (*r* = 0.28, *p* = .003) in the UG_AMB_ became statistically significant, though the giving in the UG_AMB_ correlated more strongly with MAO (*r* = 0.44, *p* < .0001) or expectation (*r* = 0.31, *p* = .001) in the UG_STD_. On the other hand, elimination of those strong over-estimators of rejection hardly affected the correlation between the effect of intentionality manipulation on giving (i.e., giving in the UG_STD_−UG_AMG_) and its effect on MAO (*r* = -0.06, *p* = .524) or expectation (*r* = 0.02, *p* = .860).

### Modulation of giving by CRT

**Fig 4:**
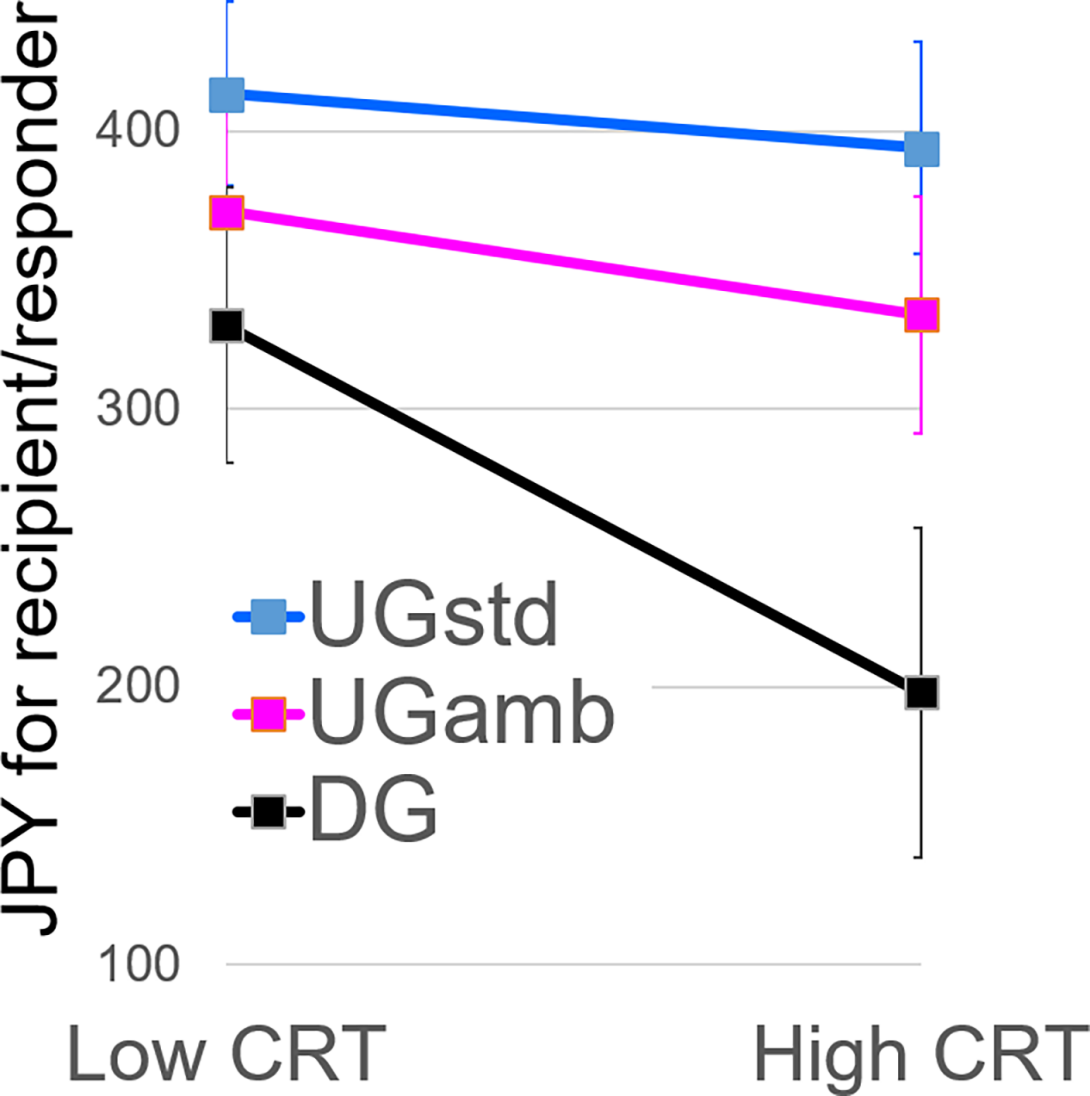
The levels of giving in the DG, the UG_STD_, and the UG_AMB_ as a function of the categorized CRT. Error bars represent 95% confidence intervals in the relevant within-participant condition.

As mentioned in the Methods section, we excluded five participants who failed to answer some of the questions of the CRT from the analysis involving the CRT (remaining n = 116). Conceptually replicating the earlier studies with the DLPFC cortical thickness [18, 26, 27], the CRT score was positively correlated with the difference in giving between the DG and the UG_STD_ (*r* = .18, *p* = .051) (Fig 1). Furthermore, the CRT score negatively correlated with giving only in the DG (*r* = -.29, *p* = .002), but not significantly with giving in the UG_STD_ (*r* = -.18, *p* = .060) or the UG_AMB_ (*r* = -.12, *p* = .212) (Fig 1B). When the participants were categorized into high CRT scorers (n = 50) who gave correct answers to all three quizzes and low CRT scorers who made at least one intuitive error (n = 66), the interaction effect of the categorized CRT and the three games in the repeated measure analysis of variance of giving was significant (*F*(2, 228) = 6.12, *p* = .003, *η*^2^ = .051). These results indicate that the previously found [18, 26, 27] difference between the DG and the UG_STD_ was caused mostly by those who could control their intuitive drive for prosociality in the DG, not in the UG_STD_ (cf., Fig 4).

We then focused our analysis on the comparison between the UG_STD_ and the UG_AMB_. In a repeated measures ANOVA of the proposer’s giving by the game type and the CRT, the main effect of game type was significant (*F*(1, 114) = 6.70, *p* = .011, *η*^2^ = .056), the main effect of the CRT was not significant (*F*(1, 114) = 1.22, *p* = .271, *η*^2^ = .011), and the game type by CRT interaction effect was not significant (*F*(1, 114) = 0.50, *p* = .483, *η*^2^ = .004). The last two results indicate that the CRT did not modulate the effect of the intention manipulation on giving.

### Modulation of MAO and anticipation of rejection by CRT

**Fig 5:**
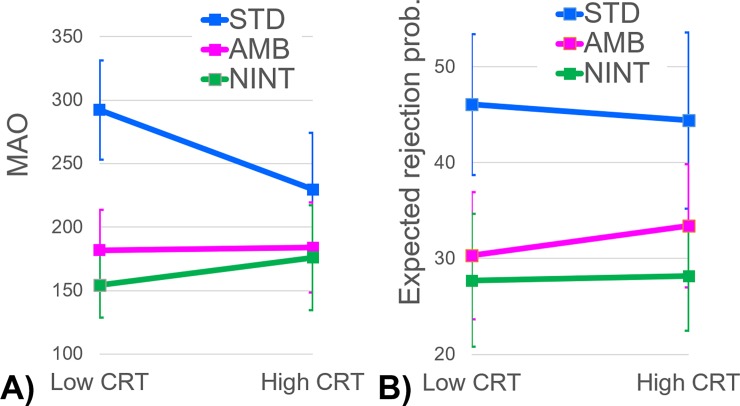
**The levels of MAO (Panel A) and expected rejection probability of an unfair offer (Panel B) in the UG**_**STD**_**, the UG**_**AMB**_**, and UG**_**NINT**_ **as functions of the categorized CRT.** Error bars represent 95% confidence intervals in the relevant within-participant condition.

We next examined if the CRT differentially affected the levels of MAO in the three UG games. The interaction effect of the CRT and the three game types was significant (*F*(2, 228) = 12.27, *p* < .0001, *η*^2^ = .097). As shown in Fig 5A, game differences in MAO were smaller for the high CRT scorers than the low CRT scorers, especially between the UG_STD_ and the other two games. The interaction effect of categorized CRT by the UG_STD_ versus the UG_AMB_ was also significant (*F*(1, 114) = 7.84, *p* = .006, *η*^2^ = .064). The two-game difference was more pronounced in the low CRT scorers (difference *M* = 110.61 ± 33.99; *F*(1,65) = 42.24, *p* < .0001, *η*^2^ = .394) than the high CRT scorers (difference *M* = 46.00 ± 28.25; *F*(1,49) = 10.71, *p* = .002, *η*^2^ = .179). That is, intentionality of the proposer had a larger effect on MAO among the low CRT scorers than the high CRT scorers, suggesting that MAO in the UG_STD_ is more strongly affected by intuitive drive for rejecting unfair offers among the former than the latter players. Comparable results were obtained regarding the anticipated rejection of unfair offers by other players (Fig 5B); the two-game difference was more pronounced in the low CRT scorers (*M* = 15.76 ± 5.72; *F*(1,65) = 30.25, *p* < .0001, *η*^2^ = .318) than the high CRT scorers (*M* = 11.00 ± 5.75; *F*(1,49) = 14.79, *p* < .001, *η*^2^ = .232), although the interaction of the categorized CRT and the anticipated rejection was not statistically significant (*F*(1, 114) = 1.33, *p* = .252, *η*^2^ = .012).

## Discussion

The primary finding of this study is that manipulation of the proposer’s second-order knowledge about his/her intentionality (I know that my partner does not know my intention) induces the proposer to give less to the recipient. This finding was made possible for the first time using the UG_AMB_, a new variant of the UG. According to the standard view of the proposer as a strategic decision maker, this finding makes perfect sense; the knowledge that the recipient is unclear about malignance of the proposer’s intention in the UG_AMB_ makes the proposer expect that unfair offers are less likely to be rejected, and accordingly, makes the proposer take a larger share of the endowment with reduced fear of being rejected. This standard view implies that the proposer’s mind reading, which partly reflects projection of their own responses (MAO) when they played the role of responder, mediates their levels of giving in the UG_AMB_.

However, an alternative, preference-based account proposed in an earlier study [26] based on the comparison of giving in the DG and the UG, is also possible. That is, scrutiny of the DG’s incentive structure especially among those who are high on cognitive control suppressed intuitive drive for prosocial choices in the DG (cf., Fig 1B). This alternative view of the proposer’s giving in the UG_AMB_ does not assume mediation of giving by their own MAO or the anticipated level of rejection by others. The two alternative predictions of the mediation role of mind reading turned out to be in favor of the alternative view of the proposer’s giving. That is, the effect of intention manipulation on giving (giving in the UG_STD_–UG_AMB_) did not correlate with the same effect on MAO (MAO in the UG_STD_–UG_AMG_) or on other players’ anticipated willingness to reject unfair offers. This result implies that the effect of the intention manipulation on giving was independent of its effect on MAO or anticipated others’ rejection likelihood, and thus the latter effect could not mediate the former.

It seemed that the players suppressed their intuitive drive for prosocial giving more in the UG_AMB_ than in the UG_STD_, instead of using their mind reading ability to infer the responder’s rejection likelihood and strategically calculating the most profitable offer more in the former than the latter game. This conclusion, however, requires caution and future study before being firmly established, because the measure of expectation of other players’ rejecting an unfair offer was not incentivized as was done in some study [40] in which players were paid for correctly guessing other players’ choices. The use of incentivized measure of expectation, however, can be a double-edged sword. On the one hand, it may reduce haphazard answers and thus help improve the measure’s reliability and validity. On the other hand, it may unwittingly induce players to focus on other people’s responses; accordingly, some participants who otherwise act intuitively may be artificially lead to act strategically. This will introduce a bias in the result in favor of a rational-strategic view of human decision making. Carefully comparing the implications of the two ways of measuring participants’ expectations—incentivized and non-incentivized—in future studies is needed.

Our second goal was also clearly achieved. We provided additional evidence that a difference in giving between those with strong and weak cognitive control existed in the DG, but not in the UG; this finding conceptually replicated the earlier findings [26, 27] that the DG–UG difference was caused by cognitive suppression of altruistic drive rather than strategic calculations based on mind reading. As suggested in the earlier studies [26, 27], the simple incentives in the one-shot DG rather than mind reading of the partner made those who were capable of cognitive control suppress their altruistic drive in the DG. The absence of mediation or modulation by the CRT in the UG_STD_–UG_AMB_ difference in giving was found to be attributable to the absence of simplicity in incentives in the UG_AMB_ compared to the DG.

One potential limitation of our study derives from the validity of CRT as an indicator of the use of deliberative assessment to override intuitive choices. For example, Welsh, Burns, and Delfabbro [41] found that CRT score is confounded with other factors such as the ability for numerical manipulations or general intelligence, which is a correlate of numeric ability. Although there is some evidence that general intelligence suppresses the level of prosocial choices in one-shot economic games [40, 42], it has not been determined yet whether the intuition controlling function is specific to the CRT-related aspect of intelligence or the general intelligence as it is measure by the IQ score. What is unique to CRT in suppressing intuitive drive for prosocial giving as well as rejection of unfair offers is an important topic for future studies.

The possibility that our findings were caused by the fixed order that our participants played the games—all participants played the games in the order of the DG, the UG_STD_, the UG_NINT_, and the UG_AMB_—needs to be examined in future research. We used this order because each variant of the UG required knowledge about the previous games. For example, it was impossible to explain the UG_AMB_ without first explaining the UG_STD_ and the UG_NINT_. Similarly, the explanation of the UG_NINT_ required that the participant had understood the UG_STD_. Although it was possible for all participants to be first informed of all the different versions of the UG before playing the games, and then assigned to the games in a counter-balanced order, this way of avoiding the order effect would have demanded a high level of participants’ cognitive effort and could have diverted the participants’ effortful attention from the differences between games when they played them. While the use of a fixed order was the only practically available method in this sense, we still need to consider the possible influence of the use of this fixed order. For example, the finding that giving in the UG_AMB_ was more strongly related with MAO in the UG_STD_ than with MAO in the same UG_AMB_ may be the consequence of playing the UG_STD_ first. That is, participants’ MAO in the UG_STD_ left a strong impression on the players, and the impression played a dominant role when they played the proposer’s role in the UG_AMB_. The potential biases caused by the fixed manipulation order need be addressed in a future study that utilizes a between-participant design.

## Supporting information

S1 Table English translation of instructions for the four games used in the study. (PDF)

S2 Table Non-parametric analysis results for the analyses reported in the main text. (DOCX)

S3 Table Analysis results with three indices of the CRT. The choice of index does not make much difference in the conclusion.
(DOCX)

S4 Table Data used for the analysis reported in the article. (XLSX)
