# Supplementary material for: Preference and strategy in proposer’s prosocial giving in the ultimatum game
Source: PLoS One. 2018 Mar 5;13(3):e0193877. doi: 10.1371/journal.pone.0193877 (PMC5837294; doi:10.1371/journal.pone.0193877)
Supplement: S1 Table — (PDF) [file pone.0193877.s001.pdf]

S1 Table

English Translation of Instructions for the Four Games Used in the Study

Preference and Strategy in Proposer's Prosocial Giving in the Ultimatum Game

Misato Inaba[1], Yumi Inoue[2], Satoshi Akutsu[3], Nobuyuki Takahashi[4], Toshio Yamagishi[3*]

[1]Center for experimental economics, Kansai University

[2]Faculty of Economics, Teikyo University

[3]Graduate School of International Corporate Strategy, Hitotsubashi University

[4]Graduate School of Letters, Hokkaido University

*Corresponding author: Toshio Yamagishi, Graduate School of International Corporate Strategy, Hitotsubashi University, 2-1-2 Hitotsubashi, Tokyo 101-8439, Japan. Email: yamagishitoshio@gmail.com

General Instruction	
本日はご協力いただき、ありがとうございます<br>• 着席されましたら、携帯電話の電源をお切りください。<br>• 以下のご記入をボールペンでお願いいたします。<br>文字は大きく丁寧に記入してください。<br><br>1. 実験参加への同意書への記入<br>2. 領収書への記入<br>金額は、すべての作業が終了した後にこちらで記入しますので、氏名等のご記入をお願いします。<br><br>• 記入が終わりましたら、机の上にある空の封筒に同意書と領収書を入れてください。後ほど回収します。	Thank you for your participation today.<br>Please turn-off your cellphone as you take a seat.<br>Please fill in the following documents with a ball-point pen.<br>Please write the letters big and clearly.<br><br>1. Consent for participating in the experiment<br>2. Receipt. Fill in your names and so on. We will add the amount of payment later.<br>Please put the consent form and the receipt in an empty envelope placed on your desk. We will collect it later.
**本実験について**	About the experiment
• これから皆さんには、他の参加者とペアになり、いくつかの作業を行っていただきます。<br>• 毎回作業ごとにペア相手は変わります。毎回誰と誰がペアになるかはお知らせしません。<br>• ペアで行う作業の結果に応じて、それぞれの作業では、0円から1000円のお金を得ることができます。<br>• 参加者の人数が奇数の場合には、1人の参加者が2回ペアリングされます。ただし、2回ペアリングされた人についても、獲得金額の計算に反映されるのは、そのうちどちらか1回の作業の分だけです。	You are paired with another participant and engage in several tasks.<br>In each task, you are paired with a different participant. We will not inform you who is paired with whom.<br>Depending on the outcome of the task conducted in the pair, you will be able to earn between 0 and 1000 yen in each task.<br>When there is an odd number of participants in the session, one participant is paired with two other participants. However, only the outcome of one of the two partners will be reflected in the earning of that participant.

・獲得金額がもらえる可能性がある作業はいくつかあります。ただし、実際にお金がもらえるのは、そのなかの2つの作業のみです。<br>・実際にお金がもらえる作業がどれになるかは、すべての作業が終了したあとでくじ引きで決めます。<br>・くじ引きで選ばれた作業での獲得金額の合計（0円～2000円）に、実験参加の最低保証金額である500円を加えた合計金額を、実験終了後に現金でお渡しします。	There are several tasks of which outcomes are potentially paid to you. However, we will actually pay you only for the outcomes of two of the tasks.<br>Which two tasks are used for payment will be decided by a lottery when all tasks are completed.<br>You will be paid in cash when the experiment is over the sum of the payments you earned in the two tasks (between 0 and 2,000 yen) plus the minimum guarantee of 500 yen.
**匿名性の保証**<br>・これから行っていただく作業でのあなたの決定は、ペアの相手を含め、誰にも分からないようになっています。<br>・データはすべてID番号を使って処理されます。データの分析をする人は、ID番号しか知りません。<br>・受付の人は名前とID番号の対応は分かりますが、その人の決定については知らされません。<br>・以上の方法で、あなたの決定は完全に匿名性が保たれます。	Guarantee of anonymity<br>Everyone, including your partners in the tasks, cannot know about your decisiosn in the tasks.<br>All data is processed using ID numbers. The persons who analyze data know only the ID numbers.<br>The receptionist knows the correspondence between your name and your ID number, but he/she will not be informed of your decisions in the tasks. In this way, your decisions' anonymity is completely protected.
Instructions for the DG	
**分配作業の説明**<br>1	Instructios for the allocation task
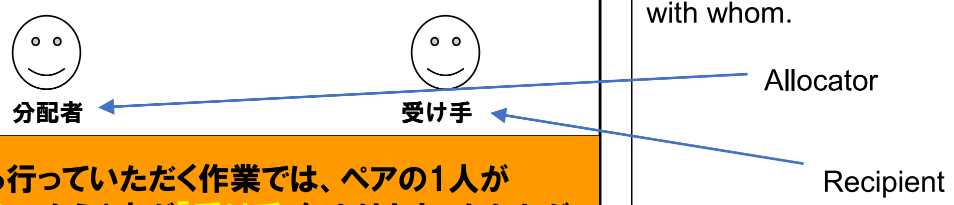<br>**これから行っていただく作業では、ペアの1人が「分配者」、もう1人が「受け手」になります。あなたがどちらの役割になるかはまだ決まっていません。誰と誰がペアになるかは、誰にもお知らせしません。**<br>2	In this task, one of each pair plays the role of a "allocator" and the other plays the role of a "recipient". It is not yet decided which role you are assigned. We will not inform you who is paired with whom.

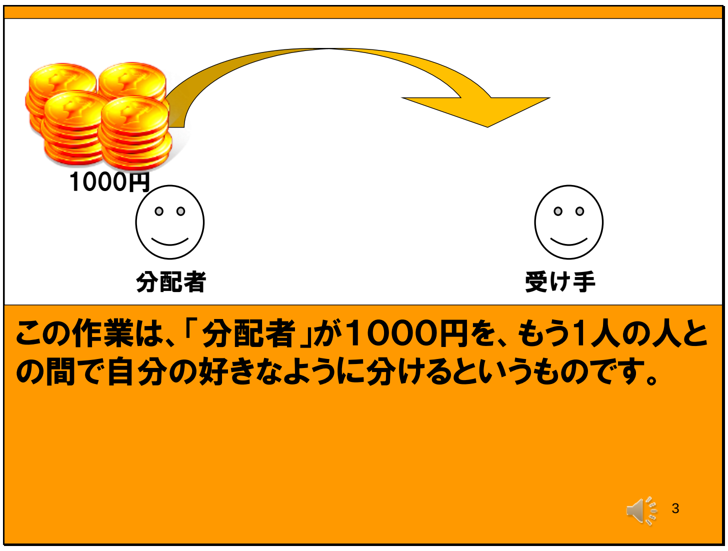

In this task, an "allocator" divides 1000 yen between him/herself and a "recipient" in any way he/she wants.

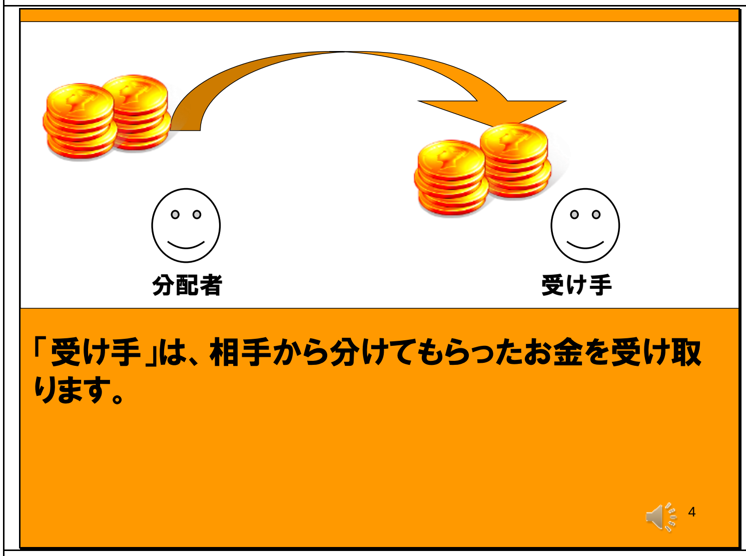

The "recipient" receives the money allocated by the “allocator”.

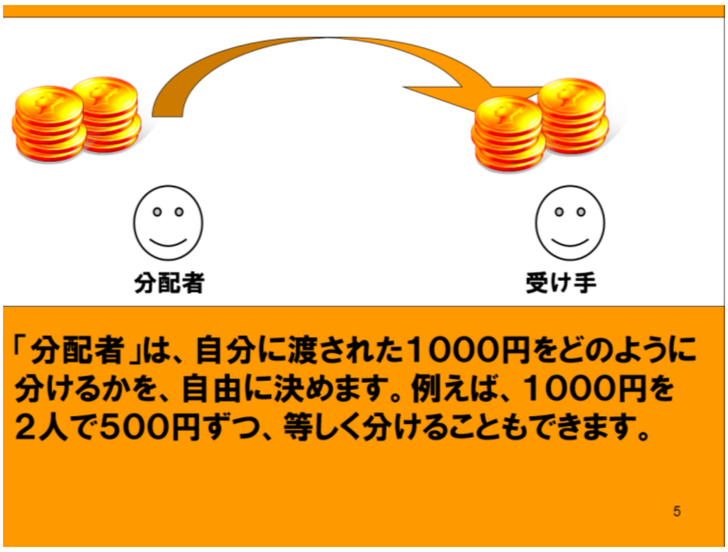

The "allocator" freely decides how to divide 1000 yen between the two. For example, he/she may divide 1000 yen equally – 500 yen for each.

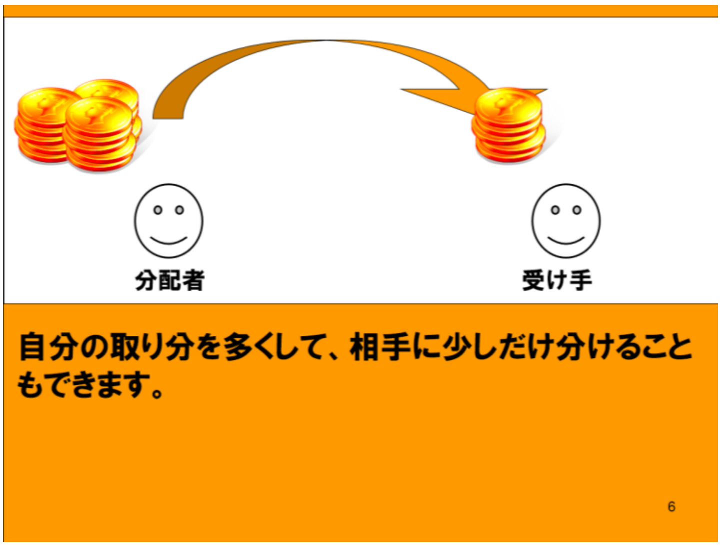

He/she may allocate more money to him/herself and less to his/her partner.

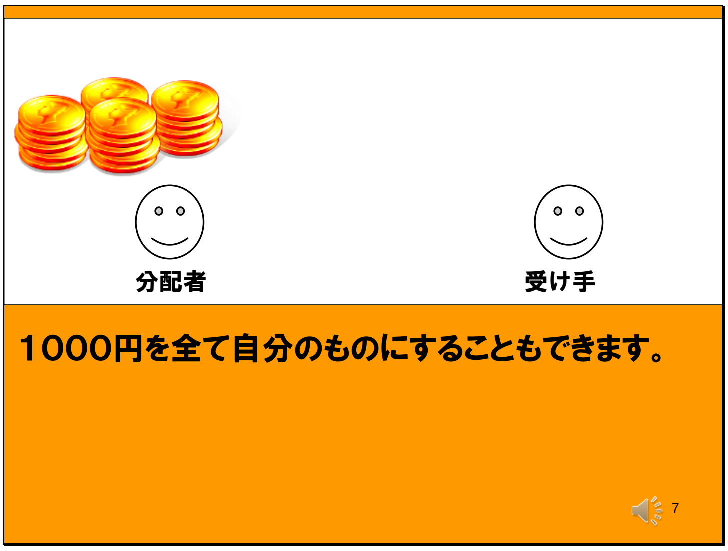

He/she may allocate all of 1000 yen to him/herself.

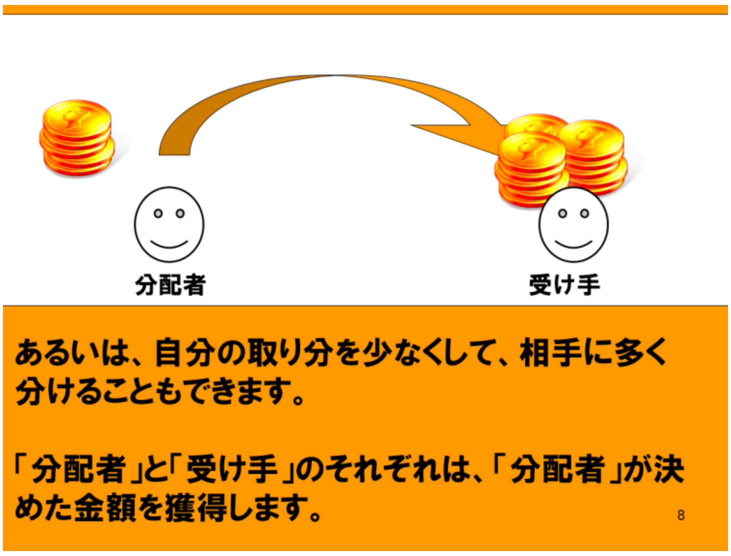

Or, he/she may allocate less to him/herself and more to his/her partner. The "allocator" and the "recipient" earn the amounts decided by the "allocator".

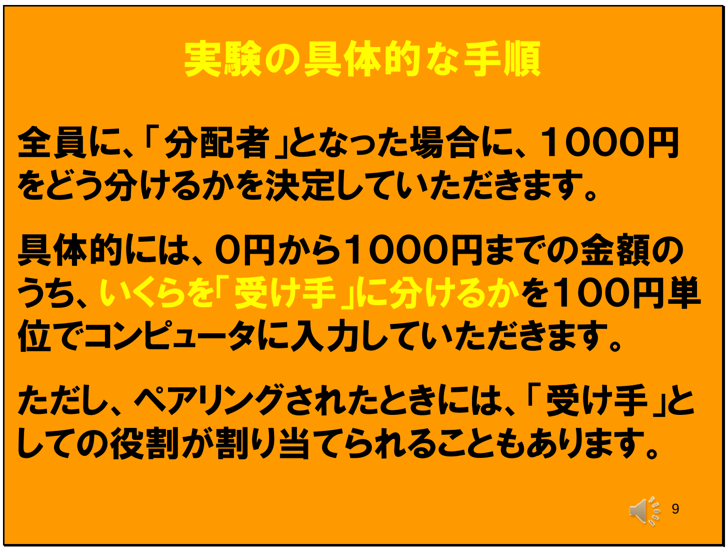

Speific procedure for the experiment

We will ask everyone to decides how to divide 1000 yen when he/she is assigned as an "allocator".

Specifically, we ask them to enter how much you will give to a "recipient" out of the amount of 0 yen to 1000 yen in inxliments of 100 yen.

Note that, some people will be assigned the role of a “recipient”

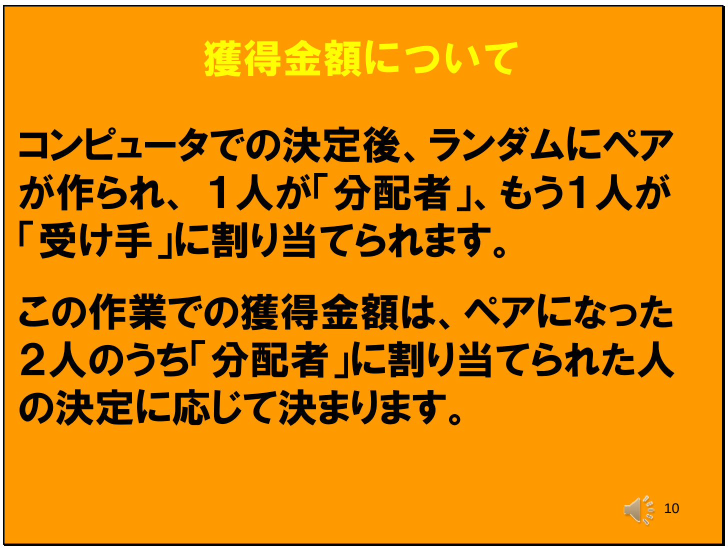

About earnings

After all people enter their decisions on the computer, pairs are formed randomly, and one of the pair is designated as an “allocator” and the other is as a “recipient”.

The earnings of the two in a pair are determined by the decision of the person who is designated as the "allocator".

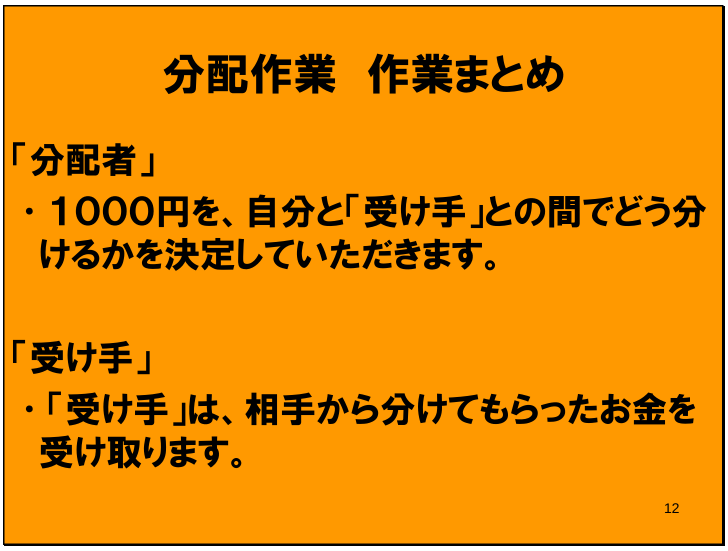

Summary of the Allocation Task

“Allocator”

As an allocator, you will decide how to divide 1000 yen between yourself and a “recipient”.

“Recipient”

As a recipient, you will receive the money allocated by your allocator.

Instructions for the UGSTD

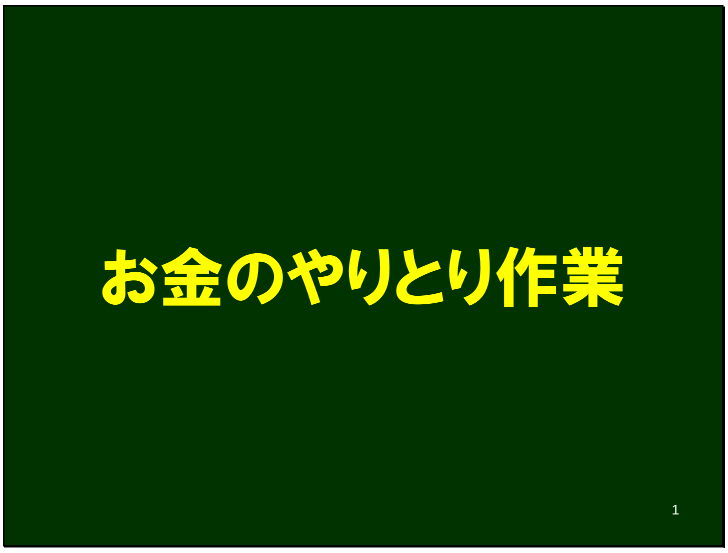

The Task of Monetary Gives and Takes

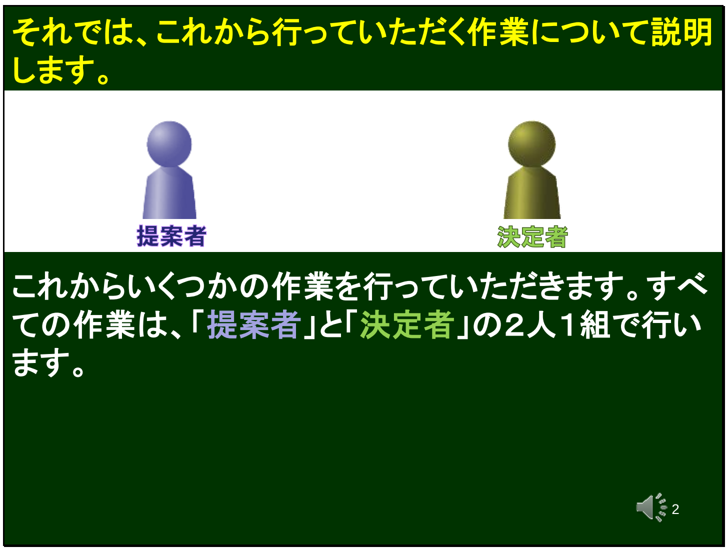

Let us explain about the task you will engage in next.

You engage in several tasks from now on.

All tasks will be conducted in pairs of a "proposer" and a "determiner".

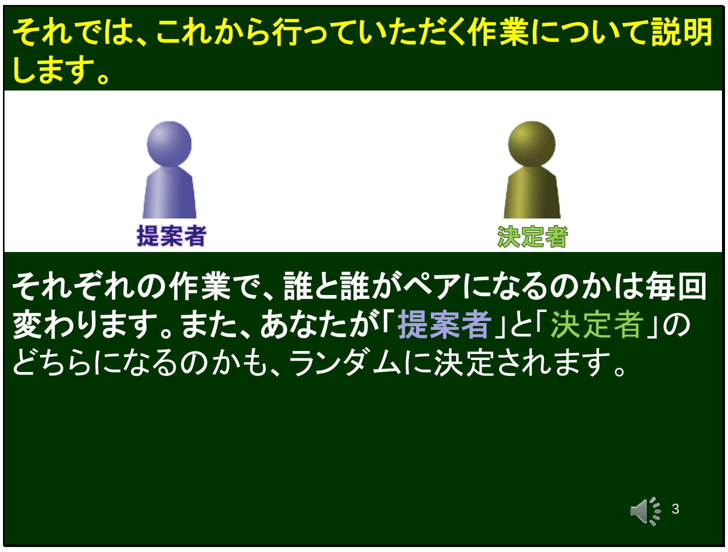

Let us explain about the task you will engage in next.

Who is paired with whom changes in each task.

Also, you will be randomly designated as a “proposer” or a “determiner”.

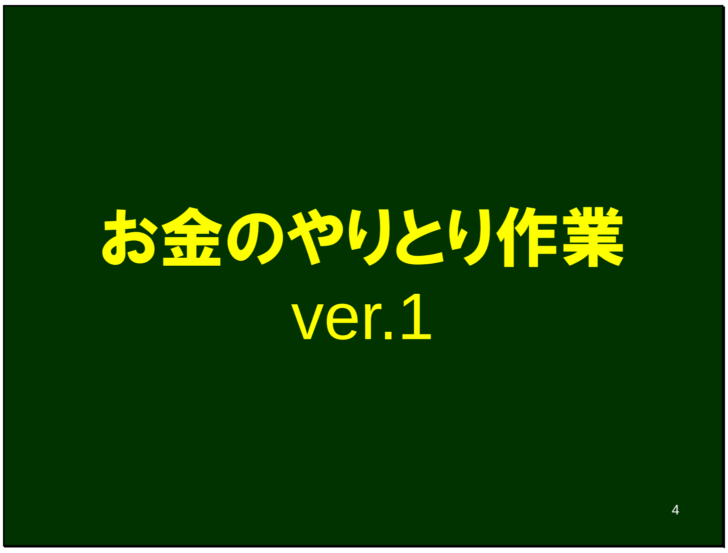

The Task of Monetary Gives and Takes, Version 1

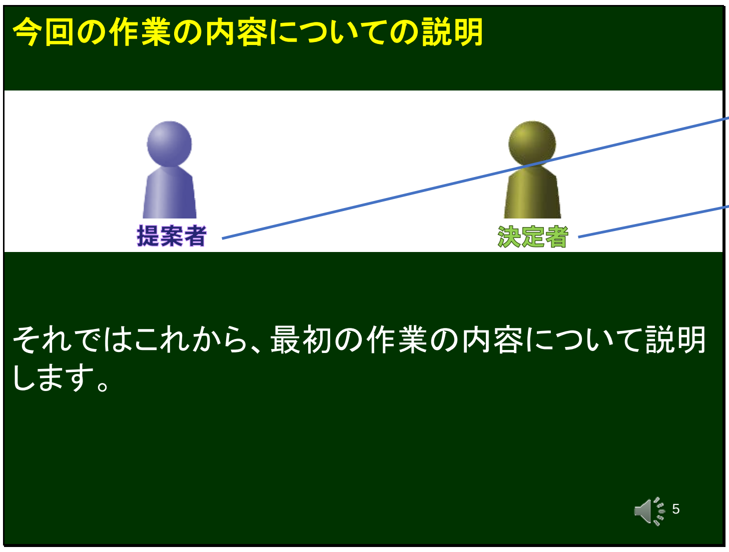

Explanation of the First Task

Proposer

Determiner

Below, we will explain about the first task.

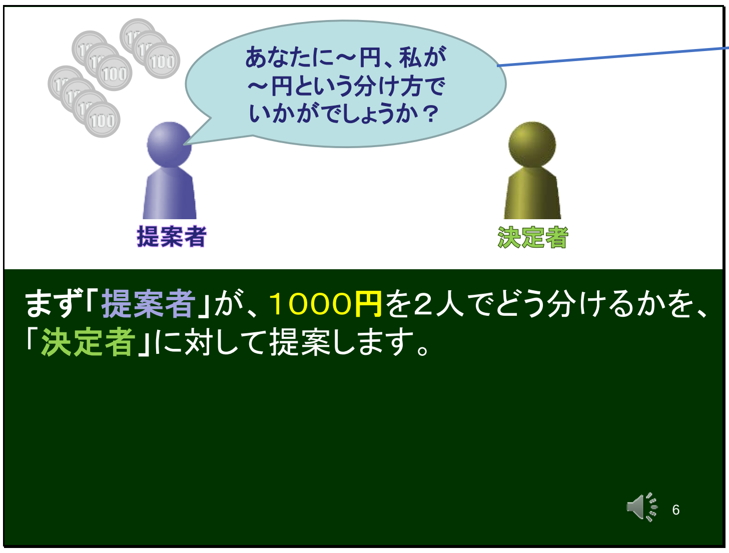

“How about my taking ~ yen and your taking ~ yen?”

First, the “proposer” proposes to the “determiner” a division of 1000 yen between the two.

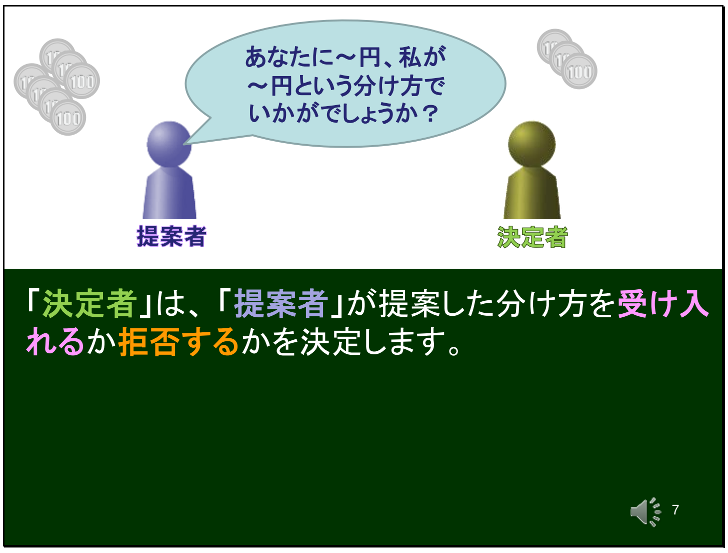

The "determiner" decides whether to accept or reject the “proposer’s” proposal for a division of money.

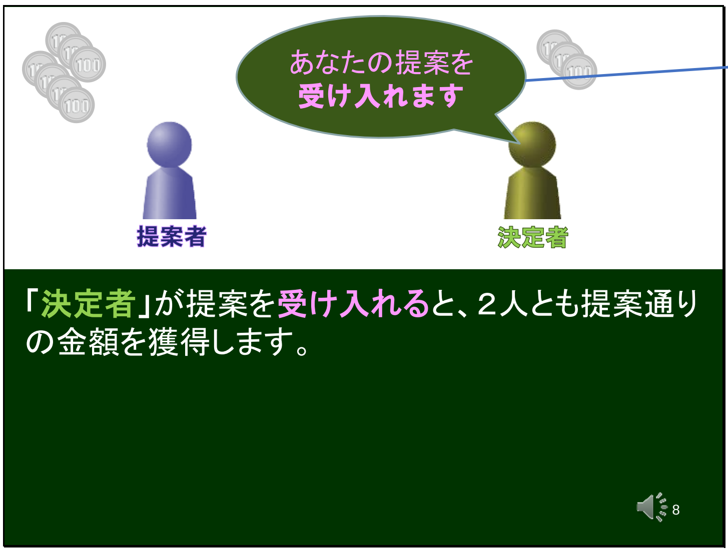

"I accept your proposal".

If the "determiner" accepts the proposal, both acquire the proposed amount.

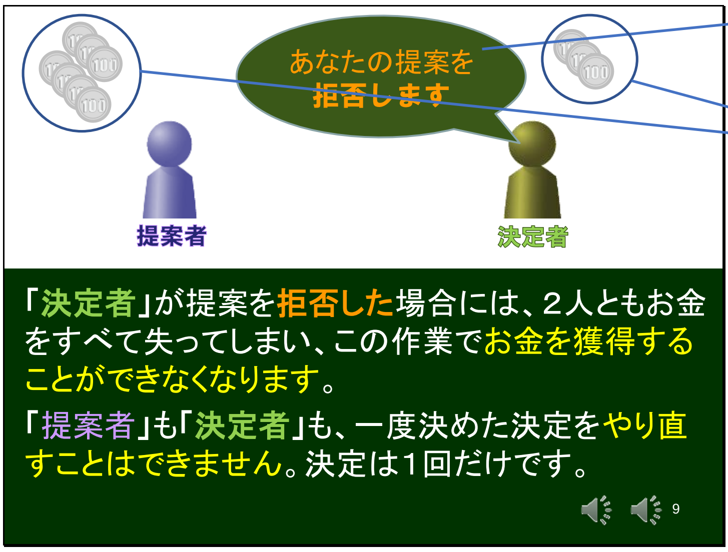

"I reject your proposal."

* These coins disappear while the participant is reading this page.

If the "determiner" rejects the proposal, both earn nothing in this task.

Neither "proposer" nor "determiner" cannot change their decisions once decided. The decision is made just once.

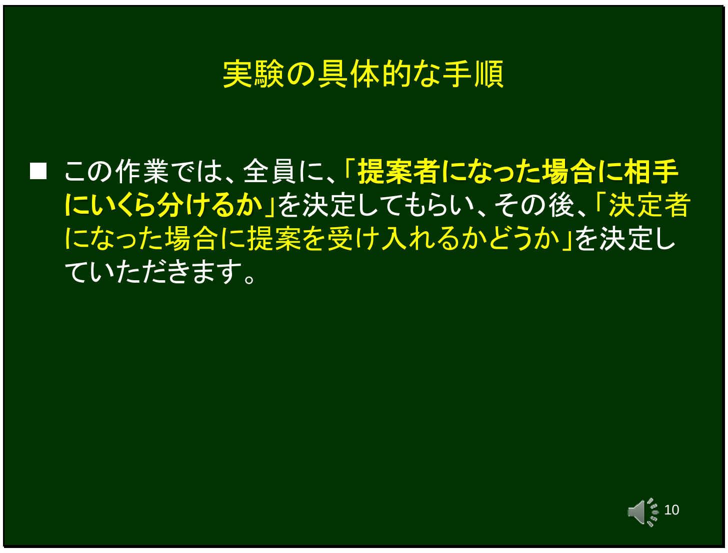

Specific Procedure for the Experiment

Everyone first decides "how to divide 1000 yen if he/she was designated as a proposer", and then decides whether or not to accept some proposals if he/she was designated as a determiner.

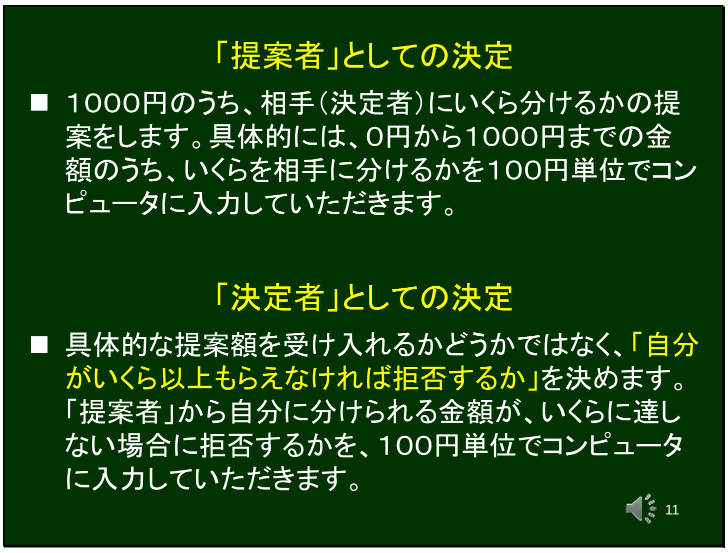

Decision as a "proposer"

You decide how much of 1000 yen you allocate to your partner (determiner). Specifically, we ask you to enter the amount that you allocate to the "determiner," in the range of 0 yen and 1000 yen in increments of 100 yen.

Decision as a "responder"

You decide the minimum amount below which you would reject any proposal, rather than deciding on specific proposals. We will ask you to enter into the computer the amount to be allocated to you below which you reject any proposal in increments of 100 yen.

獲得金額について<br>■ コンピュータでの決定後、ランダムにペアが作られ、1人が「提案者」、もう1人が「決定者」に割り当てられます。<br>■ この作業での獲得金額は、ペアになった2人の決定に応じて決まります。<br>12	About the Earnings<br>Pairs will be randomly formed when all participants completes their decisions, and one is randomly designated as a “proposer” and the other as a “determiner”.<br>How much each of the two earn is determined by the outcome of the two players’ decisions.
**お金のやりとり作業**ver.1　**作業まとめ**<br>「提案者」<br>・ １０００円を、自分と「決定者」との間でどう分けるかを決定していただきます。<br>「決定者」<br>・ 「提案者」からの提案において、自分に分けられる金額がいくらに達しなければ拒否するかを決定していただきます。<br>• ※「決定者」が拒否した場合には、この作業での「提案者」と「決定者」の獲得金額は0円になります。<br>13	Summary of the Task of Monetary Gives and Takes, Version 1<br>“Proposer”<br>As a “proposer,” you decide how to divide 1000 yen between yourself and a “determiner”.<br>“Determiner”<br>As a “determiner,” you decide the amount to be allocated to yourself below which you reject any proposal.<br>* When the “determiner” rejects the proposal, both the “proposer” and the “determiner” earn no money in this task.
Instructions for the UG_{NINT}	
**お金のやりとり作業**<br>Ver.2	The Task of Monetary Gives and Takes, Version 2
先ほどの作業と異なる点<br>■ 先ほどの作業では、「提案者」は、自分と「決定者」との間で１０００円をどう分けるかを決めて、その分け方を提案することになっていました。<br>■ しかし今度の作業では、「提案者」は分け方を**自由に決めることができません**。	How This Task differs from the Previous One<br>In the previous task, the “proposer” proposed how to divide 1000 yen between him/herself and a “determiner”.<br>However, in this new task, “proposer” cannot freely decide how to divide the money.

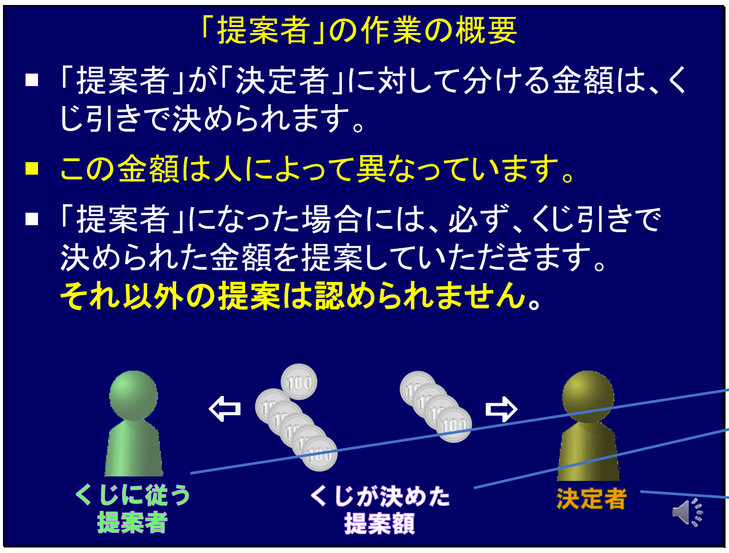

Overview of the "Proposer's" Task

How much the "proposer" allocate to the "determiner" is determined by a lottery.

This amount is different for each "proposer".

If you are designated as a proposer, you ought to propose the amount decided by the lottery. It is the only proposal you are allowed to make.

Proposer who follows the lottery

Allocation of money determined by a lottery

Determiner

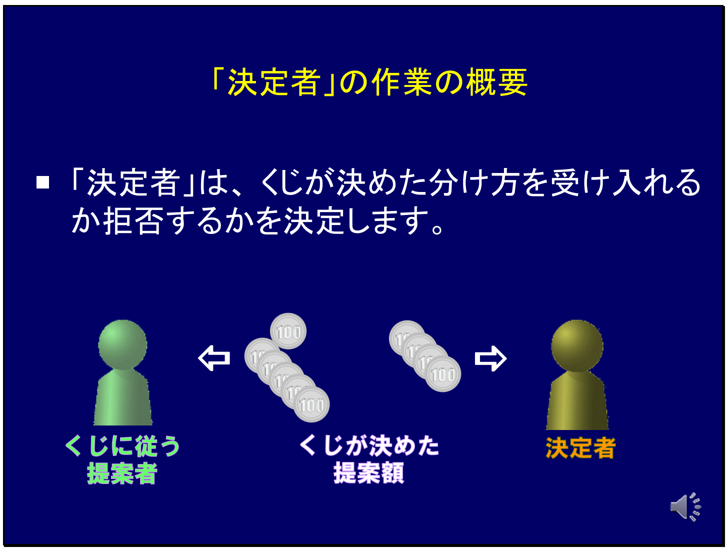

Overview of the "Determiner's" Task

The "determiner" decides whether to accept or reject the proposal which has been decided by a lottery.

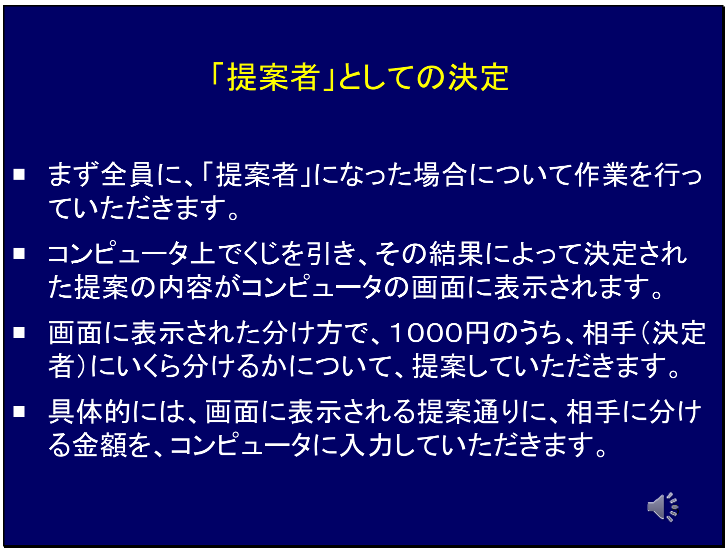

Decision as a "proposer"

We first ask all of you to perform the task as a "proposer."

You draw a lottery on the computer, and the offer made by the computer will be displayed on the computer screen.

We ask you to propose to the determiner how much of JPY 1,000 to allocate to the responder according to the outcome of the lottery shown on the display

Specifically, we will ask you to enter into the computer the monetary value to be allocated to the determiner, which is displayed on the screen.

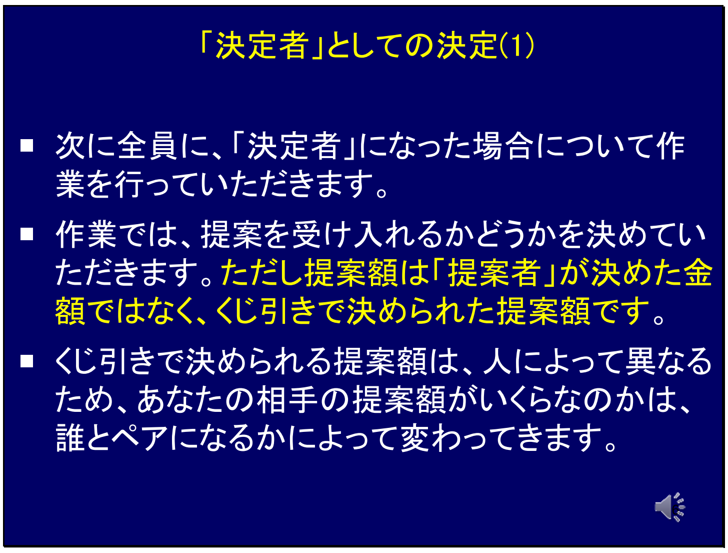

Decision as a "Determiner", 1

Next, we will ask you to perform the "determiner's" task.

In this task, you are asked to decide whether to accept or not the proposal. However, the monetary value offered to you is not the value that the proposer determined. It is the value determined by a lottery.

The offered value determined by the lottery varies from person to person, and thus how much you will be offered changes who you are paired with.

「決定者」としての決定(2)<br>■ 具体的には、くじ引きで決められた提案額を受け入れるかどうかではなく、「自分がいくら以上もらえなければ拒否するか」を決めます。<br>■ 「提案者」から分けられる金額が、いくらに達しない場合に拒否するかを、100円単位でコンピュータに入力していただきます。	Decision as a “determiner”, 2<br>You decide the minimum amount below which you would reject any proposal, rather than deciding on specific proposals.<br>We will ask you to enter into the computer the amount to be allocated to you below which you reject any proposal in increments of 100 yen.
獲得金額について<br>■ コンピュータでの決定後、ランダムにペアが作られ、1人が「提案者」、もう1人が「決定者」に割り当てられます。<br>■ この作業での獲得金額は、ペアになった2人の決定に応じて決まります。	About the Earnings<br>Pairs will be randomly formed when all participants complete their decisions, and one is randomly designated as a “proposer” and the other as a “determiner”.<br>How much each of the two earn is determined by the outcome of the two players’ decisions.
**お金のやりとり作業**ver.2 **作業まとめ**<br>「提案者」<br>・ くじを引いて、その結果通りに、1000円を、自分と「決定者」との間でどう分けるかを入力していただきます。<br>「決定者」<br>・ 「提案者」からの提案において、自分に分けられる金額がいくらに達しなければ拒否するかを決定していただきます。<br>※「決定者」が拒否した場合には、この作業での「提案者」と「決定者」の獲得金額は0円になります。	Summary of the Task of Monetary Gives and Takes, Version 2<br>“Proposer”<br>As a “proposer,” you draw a lottery, and propose a division of 1000 yen between yourself and a “determiner” as indicated by the lottery outcome.<br>“Determiner”<br>As a “determiner,” you decide the amount to be allocated to yourself below which you reject any proposal.<br>* When the “determiner” rejects the proposal, both the “proposer” and the “determiner” earn no money in this task.
Instructions for the UG_{AMB}	
**お金のやりとり作業**<br>Ver.3<br>1	The Task of Monetary Gives and Takes, Version 3

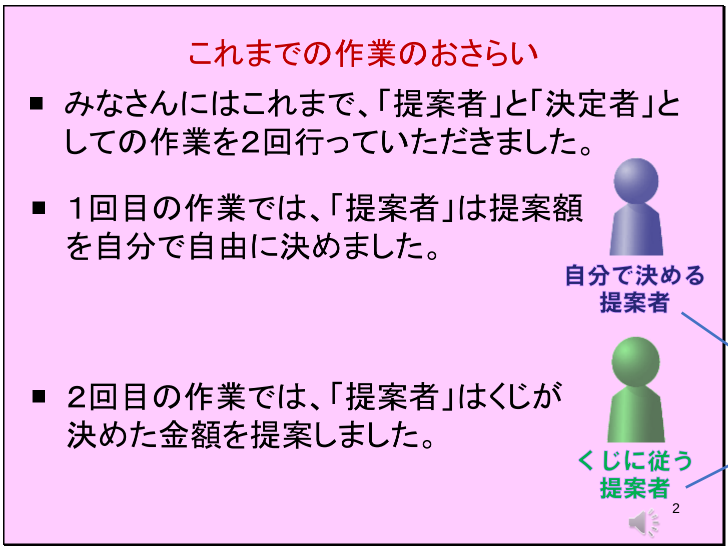

Review of the Previous Tasks

You have performed two tasks that involved the roles of “proposer” and “determiner”.

In the first task, the “proposer” proposed the division of money that he/she decided.

In the second task, the “proposer” proposed the division of money determined by a lottery.

Proposer who decides on his/her own.

Proposer who follows the decision made by a lottery

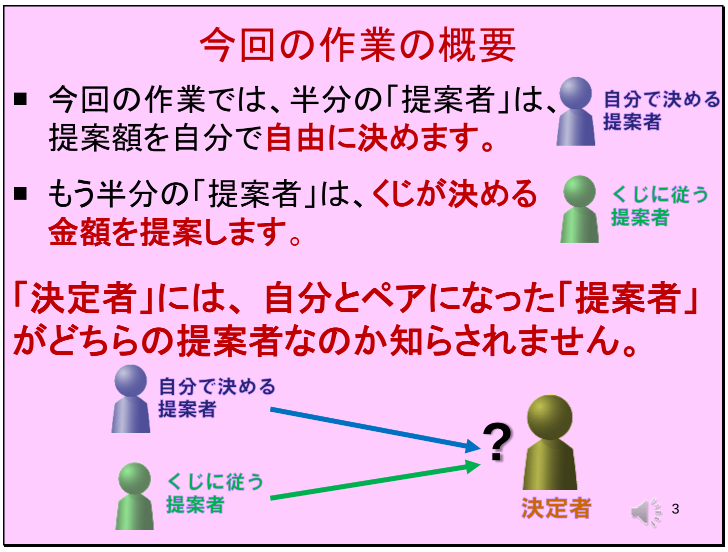

Overview of the Current Task

In the current task, half of the proposers proposes a division of money they themselves decided.

The other half propose the division of money determined by a lottery.

“Determinants” will not be informed if the proposer paired with them is which type of proposer.

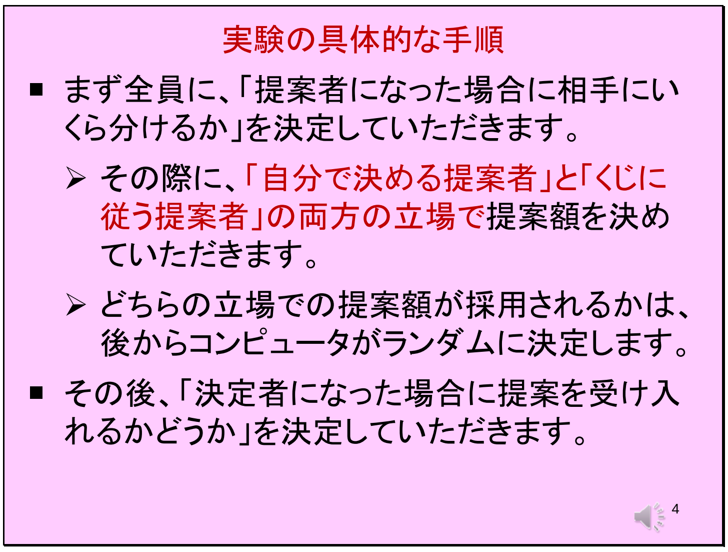

Specific Procedure for the Experiment

First, everyone decides how much to allocate to the partner when he/she is designated as a "proposer".

He/she makes the proposal both in the case he/she is designated as a proposer who makes his/her own decision and as a proposer who follows the outcome of a lottery.

Which proposal is actually used is randomly determined later by the computer.

Then, everyone makes a decision whether they would accept or reject a proposal when he/she is designated as a determiner.

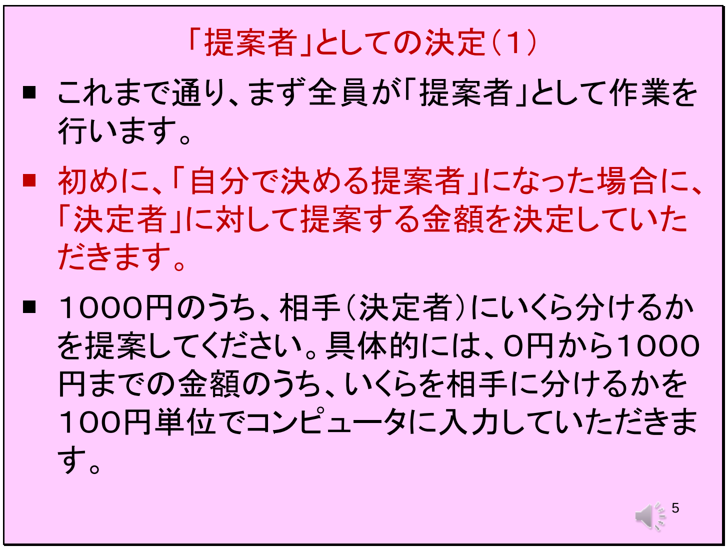

Decision as a “Proposer”, 1

As was done in the previous tasks, everyone makes a decision as a "proposer".

First, you make an allocation decision as a “proposer” who makes own decision. That is, you decide how much you would allocate to a “determiner”

You make a proposal of how much of 1000 yen to allocate to your partner (determiner). Specifically, we ask you to enter an amount that you give to your partner in the range of 0 yen to 1000 yen in increments of 100 yen.

「提案者」としての決定(2)<br>■ 続いて、「くじに従う提案者」になった場合に、「決定者」に対して提案する金額を決定していただきます。<br>■ くじを引き、画面に表示された分け方で、1000円のうち、相手(決定者)にいくら分けるかについて、提案していただきます。<br>■ 具体的には、画面に表示される提案通りに、相手に分ける金額を、コンピュータに入力していただきます。<br>6	Decision as a "Proposer", 2<br>Next, you make a proposal, as a "proposer who follows the outcome of a lottery", of how much to allocate to a determiner.<br>You draw a lottery on the computer, and propose to the responder how much of JPY 1,000 to allocate to your partner (determiner) according to the outcome of the lottery shown on the display<br>Specifically, we will ask you to enter into the computer the monetary value to be allocated to the determiner, which is displayed on the screen.
「決定者」としての決定<br>■ 次に、「決定者」としての作業を行っていただきます。<br>■ 作業では、提案を受け入れるかどうかを決めていただきます。その提案額が、「自分で決める提案者」と、「くじに従う提案者」のどちらの立場で決められた金額なのかは「決定者」にはわかりません。<br>■ 具体的には、提案額を受け入れるかどうかではなく、「自分がいくら以上分けてもらえなければ拒否するか」を決めます。<br>「提案者」から分けられる金額が、いくらに達しない場合に拒否するかを、100円単位でコンピュータに入力していただきます。<br>7	Decision as a "Determiner", 1<br>Next, we will ask you to perform the "determiner's" task.<br>In this task, you are asked to decide whether to accept or not the proposal. Note that you will not know if the allocated amount shown to you is the amount made by the proposer who makes own decision or it is the amount shown by a proposer who follow the outcome of a lottery.<br>Specifically, you decide the minimum amount below which you would reject any proposal, rather than deciding on specific proposals.<br>We will ask you to enter into the computer the amount to be allocated to you below which you reject any proposal in increments of 100 yen.
獲得金額について<br>■ コンピュータでの決定後、ランダムにペアが作られ、1人が「提案者」、もう1人が「決定者」に割り当てられます。<br>■ 「提案者」に割り当てられた場合には、「自分で決める提案者」と「くじに従う提案者」のどちらかの立場での提案が、ランダムに採用されます。<br>■ この作業での獲得金額は、ペアになった2人の決定に応じて決まります。<br>8	About the Earnings<br>Pairs will be randomly formed when all participants complete their decisions, and one is randomly designated as a "proposer" and the other as a "determiner".<br>If you are designated as a "proposer", one of your decision, as a proposal made by yourself or by a lottery, is randomly adopted as your proposal.<br>How much each of the two earn is determined by the outcome of the two players' decisions.

お金のやりとり作業ver.3 作業まとめ<br>「提案者」<br>「自分で決める提案者」 自分で決める提案者<br>→1000円を、どう分けるかを決定します。<br>「くじに従う提案者」 くじに従う提案者<br>→くじを引いて、その結果を入力していただきます。<br>「決定者」<br>自分に分けられる金額がいくらに達しなければ拒否するかを決定していただきます。<br>※「決定者」が拒否した場合には、この作業での「提案者」と「決定者」の獲得金額は0円になります。<br>9	Summary of the Task of Monetary Gives and Takes, Version 3<br>“Proposer”<br>“Proposer who make a decision by himself”→ You decide how to divide 1000 yen.<br>“Proposer who follows the outcome of a lottery→ You draw a lottery, and enter the outcome of the lottery to the computer.<br>“Determiner”<br>As a “determiner,” you decide the amount to be allocated to yourself below which you reject any proposal.<br>* When the “determiner” rejects the proposal, both the “proposer” and the “determiner” earn no money in this task.
