# Supplementary material for: Preference and strategy in proposer’s prosocial giving in the ultimatum game
Source: PLoS One. 2018 Mar 5;13(3):e0193877. doi: 10.1371/journal.pone.0193877 (PMC5837294; doi:10.1371/journal.pone.0193877)
Supplement: S2 Table — (DOCX) [file pone.0193877.s002.docx]

**S2 Table**

**Non-parametric Analysis Results for the Analyses Reported in the Main Text**

Preference and Strategy in Proposer’s Prosocial Giving in the Ultimatum Game

Misato Inaba^1^, Yumi Inoue^2^, Satoshi Akutsu^3^, Nobuyuki Takahashi^4^, Toshio Yamagishi^3*^

^1^Center for experimental economics, Kansai University

^2^Faculty of Economics, Teikyo University

^3^Graduate School of International Corporate Strategy, Hitotsubashi University

^4^Graduate School of Letters, Hokkaido University

*Corresponding author: Toshio Yamagishi, Graduate School of International Corporate Strategy, Hitotsubashi University, 2-1-2 Hitotsubashi, Tokyo 101-8439, Japan. Email: [yamagishitoshio@gmail.com](mailto:yamagishitoshio@gmail.com)

| Main Text | Non-parametric result |
| --- | --- |
| *F*(2, 240) = 51.71, *p* < .0001, *η*^2^ = .301 | *χ*^2^(2) = 79.17, *p*<.001 (Friedmann test) |
| *F*(1,120) = 66.20, *p* < .0001, *η*^2^ = .356 | *χ*^2^(1) = 51.57, *p*<0.001 (Friedmann test); Bonferroni corrected alpha for the overall alpha of .05 = .017 |
| *F*(1,120) = 51.16, *p* < .0001, *η*^2^ = .299 | *χ*^2^(1) = 40.70, *p*<0.001 (Friedmann test); Bonferroni corrected alpha for the overall alpha of .05 = .017 |
| *F*(1,120) = 7.43, *p* = .007, *η*^2^ = .058 | *χ*^2^(1) = 8.00, *p*=.005 (Friedmann test); Bonferroni corrected alpha for the overall alpha of .05 = .017 |
| *F*(2, 240) = 31.27, *p* < .0001, *η*^2^ = .207 | *χ*^2^(2 ) = 43.22, *p*<.001 (Friedmann test) |
| *F*(1,120) = 19.04, *p* < .0001, *η*^2^ = .137 | *χ*^2^(1) = 16.36, *p*<.0001 (Friedmann test); Bonferroni corrected alpha for the overall alpha of .05 = .017 |
| *F*(1, 120) = 15.72, *p* = .0001, *η*^2^ = .116 | *χ*^2^(1) = 17.07, *p*<.0001 (Friedmann test); Bonferroni corrected alpha for the overall alpha of .05 = .017 |
| *r* = .46, *p* < .0001 | *r* = .38, *p* < .0001 (Spearman rank-correlation) |
| *r* = .38, *p* < .0001 | *r* = .29, *p* = .001 (Spearman rank-correlation) |
| *r* = .17, *p* = .063 | *r* = .22, *p* = .016 (Spearman rank-correlation) |
| *r* = .41, *p* < .0001 | *r* = .40, *p* < .0001 (Spearman rank-correlation) |
| *z* = 2.04, *p* = 0.042 | *z* = 3.44, *p* < .001 |
| *r* = .30, *p* = .001 | *r* = .32, *p* < .0001 (Spearman rank-correlation) |
| *r* = .24, *p* = .007 | *r* = .32, *p* < .0001 (Spearman rank-correlation) |
| *z* = 0.44, *p* = 0.663 | *z* = 0.03, *p* = .972 |
| *r* = -.09, *p* = .343 | *r* = -.04, *p* = .698 (Spearman rank-correlation) |
| *r* = .01, *p* = .913 | *r* = .01, *p* = .925 (Spearman rank-correlation) |
| *r* = 0.20, *p* = .032 | *r* = 0.23, *p* = .016 (Spearman rank-correlation) |
| *r* = 0.28, *p* = .003 | *r* = 0.33, *p* < .001 (Spearman rank-correlation) |
| *r* = 0.44, *p* < .0001 | *r* = 0.42, *p* < .0001 (Spearman rank-correlation) |
| *r* = 0.31, *p* = .001 | *r* = 0.34, *p* < .001 (Spearman rank-correlation) |
| *r* = -0.06, *p* = .524 | *r* = -0.03, *p* = .756 (Spearman rank-correlation) |
| *r* = 0.02, *p* = .860 | *r* = -0.02, *p* = .982 (Spearman rank-correlation) |
| *r* = .18, *p* = .051 | *r* = .23, *p* = .012 (Spearman rank-correlation) |
| *r* = -.29, *p* = .002 | *r* = -.31, *p* = .001 (Spearman rank-correlation) |
| *r* = -.18, *p* ­= .060 | *r* = -.18, *p* ­= .057 (Spearman rank-correlation) |
| *r* = -.12, *p* ­= .212 | *r* = -.11, *p* ­= .226 (Spearman rank-correlation) |
| *F*(2, 228) = 6.12, *p* = .003, *η*^2^ = .051 | *F*(2, 228) = 6.56, *p* = .002 (GLMM*) |
| *F*(1, 114) = 6.70, *p* = .011, *η*^2^ = .056 | *F*(1, 114) = 6.67, *p* = .011 (GLMM*) |
| *F*(1, 114) = 1.22, *p* = .271, *η*^2^ = .011 | *F*(1, 106) = 1.47, *p* = .227 (GLMM*) |
| *F*(1, 114) = 0.50, *p* = .483, *η*^2^ = .004 | *F*(1, 114) = 0.39, *p* = .534 (GLMM*) |
| *F*(2, 228) = 12.27, *p* < .0001, *η*^2^ = .097 | *F*(2, 228) = 6.75, *p* = .001 (GLMM*) |
| *F*(1, 114) = 7.84, *p* = .006, *η*^2^ = .064 | *F*(1, 114) = 7.30, *p* = .008 (GLMM*) |
| *F*(1,65) = 42.24, *p* < .0001, *η*^2^ = .394 | *χ*^2^(1) = 30.42, *p*<.0001 (Friedmann test); Bonferroni corrected alpha for the overall alpha of .05 = .008 |
| *F*(1,49) = 10.71, *p* = .002, *η*^2^ = .179 | *χ*^2^(1) = 9.94, *p* =.002 (Friedmann test); Bonferroni corrected alpha for the overall alpha of .05 = .008 |
| *F*(1,65) = 30.25, *p* < .0001, *η*^2^ = .318 | *χ*^2^(1) = 21.43, *p* <.0001 (Friedmann test); Bonferroni corrected alpha for the overall alpha of .05 = .008 |
| *F*(1,49) = 14.79, *p* < .001, *η*^2^ = .232 | *χ*^2^(1) = 13.33, *p* <.0001 (Friedmann test); Bonferroni corrected alpha for the overall alpha of .05 = .008 |
| *F*(1, 114) = 1.33, *p* = .252 | *F*(1, 114) = 1.60, *p* = .208 (GLMM*) |

* We conducted GLMM analysis with a cumulative link assuming the response variable follows the multinomial distribution.
