# Supplementary material for: Preference and strategy in proposer’s prosocial giving in the ultimatum game
Source: PLoS One. 2018 Mar 5;13(3):e0193877. doi: 10.1371/journal.pone.0193877 (PMC5837294; doi:10.1371/journal.pone.0193877)
Supplement: S3 Table — The choice of index does not make much difference in the conclusion. (DOCX) [file pone.0193877.s003.docx]

## S3 Table

## Analysis Results with three Indices of the CRT

The choice of index does not make much difference in the conclusion.

##

Preference and Strategy in Proposer’s Prosocial Giving in the Ultimatum Game

Misato Inaba^1^, Yumi Inoue^2^, Satoshi Akutsu^3^, Nobuyuki Takahashi^4^, Toshio Yamagishi^3*^

^1^Center for experimental economics, Kansai University

^2^Faculty of Economics, Teikyo University

^3^Graduate School of International Corporate Strategy, Hitotsubashi University

^4^Graduate School of Letters, Hokkaido University

*Corresponding author: Toshio Yamagishi, Graduate School of International Corporate Strategy, Hitotsubashi University, 2-1-2 Hitotsubashi, Tokyo 101-8439, Japan. Email: [yamagishitoshio@gmail.com](mailto:yamagishitoshio@gmail.com)

| Index 1: Number of correct answers minus number of intuitive answersOwn MAO and the anticipated rejection likelihood of unfair offers in the UG_NINT_ or the UG_AMB_ are lower than those in the UG_STD_ Fig 2 displays the cumulative distribution of participants’ own MAO when they played the role of the responder. The main effect of the three games on the mean MAO (not including DG in which the recipient did not have the rejection option) was highly significant (*F*(2, 240) = 51.71, *p* < .0001, *η*^2^ = .301). MAO in the UG_STD_ was higher than that in the UG_NINT_ (difference *M* = 100.83 ± 24.54, 95% confidence interval), *F*(1,120) = 66.20, *p* < .0001, *η*^2^ = .356) or the UG_AMB_ (difference *M* = 81.82 ± 22.65, *F*(1,120) = 51.16, *p* < .0001, *η*^2^ = .299). The latter difference provides us with a foundation to expect more unfair offers by the proposer in the UG_AMB_ than in the UG_STD_. The difference in the mean MAO between the last two games in which the intention of the proposer was absent (UG_NINT_) or ambiguous (UG_AMB_) was relatively minor, though statistically significant (difference *M* = 19.01 ± 13.81, *F*(1,120) = 7.43, *p* = .007, *η*^2^ = .058). Giving in the UG_AMB_ is Lower than that in the UG_STD_ Fig 3 shows the cumulative distribution of giving in the three games (DG, UG_STD_, and UG_AMB_) excluding UG_NINT_ in which participants did not make the giving decision. The main effect of the game was highly significant (*F*(2, 240) = 31.27, *p* < .0001, *η*^2^ = .207). Giving in the UG_AMB_ fell in-between giving in the DG (difference *M* = 84.30 ± 38.25, *F*(1,120) = 19.04, *p* < .0001, *η*^2^ = .137) and giving in the UG_STD_ (the difference *M* = 47.93 ± 39.89, *F*(1, 120) = 15.72, *p* = .0001, *η*^2^ = .116). Own MAO and the Anticipated Rejection of Unfair Offers do not mediate the effect of ambiguous intention on giving in the UG_AMB_ The correlations between giving, MAO, and anticipated likelihood of others’ rejection of unfair offers in each game and across games are reported in Table 1. The level of giving as a proposer in the UG_STD_ was strongly correlated with own MAO in the same game (*r* = .46, *p* < .0001). That is, those who are intolerant of being treated unfairly behaved in a fair manner as proposers, and vice versa. In addition to MAO, we asked in the post-experimental questionnaire what percentage (in increments of 10%) of the other participants would reject a share of JPY 200 (20% of the total endowment). This measure of the anticipated rejection likelihood of an unfair offer by other players in the UG_STD_ was also significantly correlated with giving in the UG_STD_ (*r* = .38, *p* < .0001). In contrast to the high correlation between own MAO or anticipated rejection likelihood and giving in the UG_STD_, the level of giving in the UG_AMB_ was not significantly correlated with own MAO in the UG_ABM_ (*r* = .17, *p* = .063). Interestingly, however, giving in the UG_AMB_ was more strongly correlated with MAO in the UG_STD_ (*r* = .41, *p* < .0001) rather than in the same UG_AMB_; the difference in the correlation coefficients was significant (*z* = 2.04, *p* = 0.042). Similarly, giving in the UG_AMB_ was more strongly correlated with the anticipated likelihood of rejection in the UG_STD_ (*r* = .30, *p* = .001) than in the UG_AMB_ (*r* = .24, *p* = .007), although the difference was not statistically significant (*z* = 0.44, *p* = 0.660). The stronger relationship of giving in the UG_AMB_ with MAO in the UG_STD_ (sensitivity to intentionally unfair treatment) than with MAO in the same game (UG_AMB_) is inconsistent with the strategic view of lower giving in the UG_AMB_, because the MAO or anticipated rejection likelihood in the same game should be used to make strategic decisions. Furthermore, this finding provides support for the preference-based interpretation, because it suggests that the participant’s sensitivity to unfair treatment which is most effectively captured by the participant’s reaction to unfair treatment in the UG_STD_, in which the proposer’s malignant intention is transparent, plays a more important role in his/her rejection decision in the UG_AMB_.  Further evidence against the strategic interpretation comes from the absence of a significant correlation between the effect of intention manipulation on giving (giving in the UG_STD_–UG_AMB_) and the same effect on MAO (MAO in the UG_STD_–UG_AMG_) (*r* = -.09, *p* = .343) or on other players’ anticipated willingness to reject unfair offers (*r* = .01, *p* = .913). That is, the effect of the intention manipulation on giving was independent of its effect on MAO or anticipated others’ rejection likelihood, and thus the latter effect cannot mediate the former. These findings suggest that our players did not strategically use own MAO or anticipated rejection likelihood in making offers to their responders. Rather, those who prefer fairness and who were more willing to give in both UG_STD_ and UG_AMB_ were the ones who were more strongly upset by intentionally unfair treatment in the UG_STD_.  This interpretation of the above analysis results, however, requires caution because the expectation measure was not incentivized. That is, participants were not provided with an incentive to carefully assess others’ willingness to reject an unfair offer and report it in the post-experimental questionnaire. While an offer of JPY 200 or less (i.e., the cumulative MAO of JPY 300) was rejected by 57.02% of responders in the UG_STD_, the mean expected probability of rejecting an offer of JPY 200 or less was 45.37%; participants generally underestimated the rejection probability by others in the UG_STD_. On the other hand, 32.23% of responders in the UG_AMB_ and 25.62% in the UG_NINT_ rejected the share of JPY 200 or less expecting on average that 31.16% of other responders in the UG_AMB_ and 29.69% in the UG_NINT_ would reject the offer. The tendency for underestimation was not clear in those games; however, some participants substantially overestimated the probability of rejection in these two games. For example, 10 participants in the UG_AMB_ and 11 participants in the UG_NINT_ expected that 80% or more responders would reject the offer of JPY 200 or less. When these extreme over-estimators were excluded from the analysis, the correlation between giving and MAO (*r* = 0.20, *p* = .042) or expectation (*r* = 0.28, *p* = .003) in the UG_AMB_ became statistically significant, though the giving in the UG_AMB_ correlated more strongly with MAO (*r* = 0.44, *p* < .0001) or expectation (*r* = 0.31, *p* = .001) in the UG_STD_. On the other hand, elimination of those strong over-estimators of rejection hardly affected the correlation between the effect of intentionality manipulation on giving (i.e., giving in the UG_STD_ – UG_AMG_) and its effect on MAO (*r* = -0.06, *p* = .524) or expectation (*r* = 0.02, *p* = .860).  **Modulation of Giving by CRT**  As mentioned in the Methods section, we excluded five participants who failed to answer some of the questions of the CRT from the analysis involving CRT (remaining n = 116). Conceptually replicating the earlier studies with the DLPFC cortical thickness [18, 27, 28], the CRT score was positively correlated with the difference in giving between the DG and the UG_STD_ (*r* = .18, *p* = .051) (Fig. 1). Furthermore, the CRT score negatively correlated with giving only in the DG (*r* = -.29, *p* = .002), but not significantly with giving in the UG_STD_ (*r* = -.18, *p* = .060) or the UG_AMB_ (*r* = -.12, *p* = .212) (Fig. 1B). When the participants were categorized into high CRT scorers (n = 50) who gave correct answers to all three quizzes and low CRT scorers who made at least one intuitive error (n = 66), the interaction effect of the categorized CRT and the three games in the repeated measure analysis of variance of giving was significant (*F*(2, 228) = 6.12, *p* = .003, *η*^2^ = .051).  We then focused our analysis on the comparison between the UG_STD_ and the UG_AMB_. The main effect in a repeated measure AVOVA of the proposer’s giving by the game type and the CRT, the main effect of the games was significant (*F*(1, 114) = 6.70, *p* = .011, *η*^2^ = .056). The main effect of the CRT was not significant (*F*(1, 114) = 1.22, *p* = .271, *η*^2^ = .011). The game type by CRT interaction effect was not significant, either (*F*(1, 114) = 0.50, *p* = .483, *η*^2^ = .004). The last two results indicate that the CRT did not modulate the effect of the intention manipulation on giving.  **Modulation of MAO and anticipation of rejection by CRT**  We next examined if the CRT differentially affected the levels of MAO in the three UG games. The interaction effect of the CRT and the three game types was significant (*F*(2, 228) = 12.27, *p* < .0001, *η*^2^ = .097). As shown in Fig. 5A, the game differences in MAO were smaller in the high CRT scorers than in the low CRT scorers, especially the difference between the UG_STD_ and the other two games. The interaction effect of categorized CRT by the UG_STD_ versus the UG_AMB_ was also significant (*F*(1, 114) = 7.84, *p* = .006, *η*^2^ = .064). The two-game difference was more pronounced in the low CRT scorers (*M for the difference* = 110.61 ± 33.99; *F*(1,65) = 42.24, *p* < .0001, *η*^2^ = .394) than the high CRT scorers (*M* = 46.00 ± 28.25; *F*(1,49) = 10.71, *p* = .002, *η*^2^ = .179). That is, intentionality of the proposer had a larger effect on MAO among the low CRT scorers than the high CRT scorers, suggesting that MAO in the UG_STD_ is more strongly affected by intuitive drive for rejecting unfair offers among the former than the latter players. Comparable results were obtained regarding the anticipated rejection of unfair offers by other players (Fig. 5B); the two-game difference was more pronounced in the low CRT scorers (*M* = 15.76 ± 5.72; *F*(1,65) = 30.25, *p* < .0001, *η*^2^ = .318) than the high CRT scorers (*M* = 11.00 ± 5.75; *F*(1,49) = 14.79, *p* < .001, *η*^2^ = .232), although the interaction of the categorized CRT and the anticipated rejection was not statistically significant (*F*(1, 114) = 1.33, *p* = .252). | Index 2: Number of correct answersOwn MAO and the anticipated rejection likelihood of unfair offers in the UG_NINT_ or the UG_AMB_ are lower than those in the UG_STD_ Fig 2 displays the cumulative distribution of participants’ own MAO when they played the role of the responder. The main effect of the three games on the mean MAO (not including DG in which the recipient did not have the rejection option) was highly significant (*F*(2, 240) = 51.71, *p* < .0001, *η*^2^ = .301). MAO in the UG_STD_ was higher than that in the UG_NINT_ (difference *M* = 100.83 ± 24.54, 95% confidence interval), *F*(1,120) = 66.20, *p* < .0001, *η*^2^ = .356) or the UG_AMB_ (difference *M* = 81.82 ± 22.65, *F*(1,120) = 51.16, *p* < .0001, *η*^2^ = .299). The latter difference provides us with a foundation to expect more unfair offers by the proposer in the UG_AMB_ than in the UG_STD_. The difference in the mean MAO between the last two games in which the intention of the proposer was absent (UG_NINT_) or ambiguous (UG_AMB_) was relatively minor, though statistically significant (difference *M* = 19.01 ± 13.81, *F*(1,120) = 7.43, *p* = .007, *η*^2^ = .058). Giving in the UG_AMB_ is Lower than that in the UG_STD_ Fig 3 shows the cumulative distribution of giving in the three games (DG, UG_STD_, and UG_AMB_) excluding UG_NINT_ in which participants did not make the giving decision. The main effect of the game was highly significant (*F*(2, 240) = 31.27, *p* < .0001, *η*^2^ = .207). Giving in the UG_AMB_ fell in-between giving in the DG (difference *M* = 84.30 ± 38.25, *F*(1,120) = 19.04, *p* < .0001, *η*^2^ = .137) and giving in the UG_STD_ (the difference *M* = 47.93 ± 39.89, *F*(1, 120) = 15.72, *p* = .0001, *η*^2^ = .116). Own MAO and the Anticipated Rejection of Unfair Offers do not mediate the effect of ambiguous intention on giving in the UG_AMB_ The correlations between giving, MAO, and anticipated likelihood of others’ rejection of unfair offers in each game and across games are reported in Table 1. The level of giving as a proposer in the UG_STD_ was strongly correlated with own MAO in the same game (*r* = .46, *p* < .0001). That is, those who are intolerant of being treated unfairly behaved in a fair manner as proposers, and vice versa. In addition to MAO, we asked in the post-experimental questionnaire what percentage (in increments of 10%) of the other participants would reject a share of JPY 200 (20% of the total endowment). This measure of the anticipated rejection likelihood of an unfair offer by other players in the UG_STD_ was also significantly correlated with giving in the UG_STD_ (*r* = .38, *p* < .0001). In contrast to the high correlation between own MAO or anticipated rejection likelihood and giving in the UG_STD_, the level of giving in the UG_AMB_ was not significantly correlated with own MAO in the UG_ABM_ (*r* = .17, *p* = .063). Interestingly, however, giving in the UG_AMB_ was more strongly correlated with MAO in the UG_STD_ (*r* = .41, *p* < .0001) rather than in the same UG_AMB_; the difference in the correlation coefficients was significant (*z* = 2.04, *p* = 0.042). Similarly, giving in the UG_AMB_ was more strongly correlated with the anticipated likelihood of rejection in the UG_STD_ (*r* = .30, *p* = .001) than in the UG_AMB_ (*r* = .24, *p* = .007), although the difference was not statistically significant (*z* = 0.44, *p* = 0.660). The stronger relationship of giving in the UG_AMB_ with MAO in the UG_STD_ (sensitivity to intentionally unfair treatment) than with MAO in the same game (UG_AMB_) is inconsistent with the strategic view of lower giving in the UG_AMB_, because the MAO or anticipated rejection likelihood in the same game should be used to make strategic decisions. Furthermore, this finding provides support for the preference-based interpretation, because it suggests that the participant’s sensitivity to unfair treatment which is most effectively captured by the participant’s reaction to unfair treatment in the UG_STD_, in which the proposer’s malignant intention is transparent, plays a more important role in his/her rejection decision in the UG_AMB_.  Further evidence against the strategic interpretation comes from the absence of a significant correlation between the effect of intention manipulation on giving (giving in the UG_STD_–UG_AMB_) and the same effect on MAO (MAO in the UG_STD_–UG_AMG_) (*r* = -.09, *p* = .343) or on other players’ anticipated willingness to reject unfair offers (*r* = .01, *p* = .913). That is, the effect of the intention manipulation on giving was independent of its effect on MAO or anticipated others’ rejection likelihood, and thus the latter effect cannot mediate the former. These findings suggest that our players did not strategically use own MAO or anticipated rejection likelihood in making offers to their responders. Rather, those who prefer fairness and who were more willing to give in both UG_STD_ and UG_AMB_ were the ones who were more strongly upset by intentionally unfair treatment in the UG_STD_.  This interpretation of the above analysis results, however, requires caution because the expectation measure was not incentivized. That is, participants were not provided with an incentive to carefully assess others’ willingness to reject an unfair offer and report it in the post-experimental questionnaire. While an offer of JPY 200 or less (i.e., the cumulative MAO of JPY 300) was rejected by 57.02% of responders in the UG_STD_, the mean expected probability of rejecting an offer of JPY 200 or less was 45.37%; participants generally underestimated the rejection probability by others in the UG_STD_. On the other hand, 32.23% of responders in the UG_AMB_ and 25.62% in the UG_NINT_ rejected the share of JPY 200 or less expecting on average that 31.16% of other responders in the UG_AMB_ and 29.69% in the UG_NINT_ would reject the offer. The tendency for underestimation was not clear in those games; however, some participants substantially overestimated the probability of rejection in these two games. For example, 10 participants in the UG_AMB_ and 11 participants in the UG_NINT_ expected that 80% or more responders would reject the offer of JPY 200 or less. When these extreme over-estimators were excluded from the analysis, the correlation between giving and MAO (*r* = 0.20, *p* = .042) or expectation (*r* = 0.28, *p* = .003) in the UG_AMB_ became statistically significant, though the giving in the UG_AMB_ correlated more strongly with MAO (*r* = 0.44, *p* < .0001) or expectation (*r* = 0.31, *p* = .001) in the UG_STD_. On the other hand, elimination of those strong over-estimators of rejection hardly affected the correlation between the effect of intentionality manipulation on giving (i.e., giving in the UG_STD_ – UG_AMG_) and its effect on MAO (*r* = -0.06, *p* = .524) or expectation (*r* = 0.02, *p* = .860).  **Modulation of Giving by CRT**  Conceptually replicating the earlier studies with the DLPFC cortical thickness [18, 27, 28], the CRT score was positively correlated with the difference in giving between the DG and the UG_STD_ (*r* = .17, *p* = .065) (Fig. 1). Furthermore, the CRT score negatively correlated with giving only in the DG (*r* = -.26, *p* = .004), but not significantly with giving in the UG_STD_ (*r* = -.16, *p* = .087) or the UG_AMB_ (*r* = -.11, *p* = .247) (Fig. 1B). When the participants were categorized into high CRT scorers (n = 50) who gave correct answers to all three quizzes and low CRT scorers who made at least one error (n = 71), the interaction effect of the categorized CRT and the three games in the repeated measure analysis of variance of giving was significant (*F*(2, 238) = 5.87, *p* = .003, *η*^2^ = .047).  We then focused our analysis on the comparison between the UG_STD_ and the UG_AMB_. The main effect in a repeated measure AVOVA of the proposer’s giving by the game type and the CRT, the main effect of the games was significant (*F*(1, 119) = 6.23, *p* = .014, *η*^2^ = .050). The main effect of the CRT was not significant (*F*(1, 119) = 1.07, *p* = .304, *η*^2^ = .009). The game type by CRT interaction effect was not significant, either (*F*(1, 119) = 0.70, *p* = .405, *η*^2^ = .006). The last two results indicate that the CRT did not modulate the effect of the intention manipulation on giving.  **Modulation of MAO and anticipation of rejection by CRT**  We next examined if the CRT differentially affected the levels of MAO in the three UG games. The interaction effect of the CRT and the three game types was significant (*F*(2, 238) = 11.62, *p* < .0001, *η*^2^ = .089). As shown in Fig. 5A, the game differences in MAO were smaller in the high CRT scorers than in the low CRT scorers, especially the difference between the UG_STD_ and the other two games. The interaction effect of categorized CRT by the UG_STD_ versus the UG_AMB_ was also significant (*F*(1, 119) = 9.48, *p* = .003, *η*^2^ = .074). The two-game difference was more pronounced in the low CRT scorers (*M for the difference* = 107.04 ± 32.34; *F*(1,70) = 43.59, *p* < .0001, *η*^2^ = .384) than the high CRT scorers (*M* = 46.00 ± 28.25; *F*(1,49) = 10.71, *p* *=* .002, *η*^2^ = .179). That is, intentionality of the proposer had a larger effect on MAO among the low CRT scorers than the high CRT scorers, suggesting that MAO in the UG_STD_ is more strongly affected by intuitive drive for rejecting unfair offers among the former than the latter players. Comparable results were obtained regarding the anticipated rejection of unfair offers by other players (Fig. 5B); the two-game difference was more pronounced in the low CRT scorers (*M* = 16.48 ± 5.52; *F*(1,70) = 35.50, *p* < .0001, *η*^2^ = .336) than the high CRT scorers (*M* = 11.00 ± 5.75; *F*(1,49) = 14.79, *p* < .001, *η*^2^ = .232), although the interaction of the categorized CRT and the anticipated rejection was not statistically significant (*F*(1, 119) = 1.81, *p* = .182, *η*^2^ = .015). | Index 3: Three minus number of intuitive answersOwn MAO and the anticipated rejection likelihood of unfair offers in the UG_NINT_ or the UG_AMB_ are lower than those in the UG_STD_ Fig 2 displays the cumulative distribution of participants’ own MAO when they played the role of the responder. The main effect of the three games on the mean MAO (not including DG in which the recipient did not have the rejection option) was highly significant (*F*(2, 240) = 51.71, *p* < .0001, *η*^2^ = .301). MAO in the UG_STD_ was higher than that in the UG_NINT_ (difference *M* = 100.83 ± 24.54, 95% confidence interval), *F*(1,120) = 66.20, *p* < .0001, *η*^2^ = .356) or the UG_AMB_ (difference *M* = 81.82 ± 22.65, *F*(1,120) = 51.16, *p* < .0001, *η*^2^ = .299). The latter difference provides us with a foundation to expect more unfair offers by the proposer in the UG_AMB_ than in the UG_STD_. The difference in the mean MAO between the last two games in which the intention of the proposer was absent (UG_NINT_) or ambiguous (UG_AMB_) was relatively minor, though statistically significant (difference *M* = 19.01 ± 13.81, *F*(1,120) = 7.43, *p* = .007, *η*^2^ = .058). Giving in the UG_AMB_ is Lower than that in the UG_STD_ Fig 3 shows the cumulative distribution of giving in the three games (DG, UG_STD_, and UG_AMB_) excluding UG_NINT_ in which participants did not make the giving decision. The main effect of the game was highly significant (*F*(2, 240) = 31.27, *p* < .0001, *η*^2^ = .207). Giving in the UG_AMB_ fell in-between giving in the DG (difference *M* = 84.30 ± 38.25, *F*(1,120) = 19.04, *p* < .0001, *η*^2^ = .137) and giving in the UG_STD_ (the difference *M* = 47.93 ± 39.89, *F*(1, 120) = 15.72, *p* = .0001, *η*^2^ = .116). Own MAO and the Anticipated Rejection of Unfair Offers do not mediate the effect of ambiguous intention on giving in the UG_AMB_ The correlations between giving, MAO, and anticipated likelihood of others’ rejection of unfair offers in each game and across games are reported in Table 1. The level of giving as a proposer in the UG_STD_ was strongly correlated with own MAO in the same game (*r* = .46, *p* < .0001). That is, those who are intolerant of being treated unfairly behaved in a fair manner as proposers, and vice versa. In addition to MAO, we asked in the post-experimental questionnaire what percentage (in increments of 10%) of the other participants would reject a share of JPY 200 (20% of the total endowment). This measure of the anticipated rejection likelihood of an unfair offer by other players in the UG_STD_ was also significantly correlated with giving in the UG_STD_ (*r* = .38, *p* < .0001). In contrast to the high correlation between own MAO or anticipated rejection likelihood and giving in the UG_STD_, the level of giving in the UG_AMB_ was not significantly correlated with own MAO in the UG_ABM_ (*r* = .17, *p* = .063). Interestingly, however, giving in the UG_AMB_ was more strongly correlated with MAO in the UG_STD_ (*r* = .41, *p* < .0001) rather than in the same UG_AMB_; the difference in the correlation coefficients was significant (*z* = 2.04, *p* = 0.042). Similarly, giving in the UG_AMB_ was more strongly correlated with the anticipated likelihood of rejection in the UG_STD_ (*r* = .30, *p* = .001) than in the UG_AMB_ (*r* = .24, *p* = .007), although the difference was not statistically significant (*z* = 0.44, *p* = 0.660). The stronger relationship of giving in the UG_AMB_ with MAO in the UG_STD_ (sensitivity to intentionally unfair treatment) than with MAO in the same game (UG_AMB_) is inconsistent with the strategic view of lower giving in the UG_AMB_, because the MAO or anticipated rejection likelihood in the same game should be used to make strategic decisions. Furthermore, this finding provides support for the preference-based interpretation, because it suggests that the participant’s sensitivity to unfair treatment which is most effectively captured by the participant’s reaction to unfair treatment in the UG_STD_, in which the proposer’s malignant intention is transparent, plays a more important role in his/her rejection decision in the UG_AMB_.  Further evidence against the strategic interpretation comes from the absence of a significant correlation between the effect of intention manipulation on giving (giving in the UG_STD_–UG_AMB_) and the same effect on MAO (MAO in the UG_STD_–UG_AMG_) (*r* = -.09, *p* = .343) or on other players’ anticipated willingness to reject unfair offers (*r* = .01, *p* = .913). That is, the effect of the intention manipulation on giving was independent of its effect on MAO or anticipated others’ rejection likelihood, and thus the latter effect cannot mediate the former. These findings suggest that our players did not strategically use own MAO or anticipated rejection likelihood in making offers to their responders. Rather, those who prefer fairness and who were more willing to give in both UG_STD_ and UG_AMB_ were the ones who were more strongly upset by intentionally unfair treatment in the UG_STD_.  This interpretation of the above analysis results, however, requires caution because the expectation measure was not incentivized. That is, participants were not provided with an incentive to carefully assess others’ willingness to reject an unfair offer and report it in the post-experimental questionnaire. While an offer of JPY 200 or less (i.e., the cumulative MAO of JPY 300) was rejected by 57.02% of responders in the UG_STD_, the mean expected probability of rejecting an offer of JPY 200 or less was 45.37%; participants generally underestimated the rejection probability by others in the UG_STD_. On the other hand, 32.23% of responders in the UG_AMB_ and 25.62% in the UG_NINT_ rejected the share of JPY 200 or less expecting on average that 31.16% of other responders in the UG_AMB_ and 29.69% in the UG_NINT_ would reject the offer. The tendency for underestimation was not clear in those games; however, some participants substantially overestimated the probability of rejection in these two games. For example, 10 participants in the UG_AMB_ and 11 participants in the UG_NINT_ expected that 80% or more responders would reject the offer of JPY 200 or less. When these extreme over-estimators were excluded from the analysis, the correlation between giving and MAO (*r* = 0.20, *p* = .042) or expectation (*r* = 0.28, *p* = .003) in the UG_AMB_ became statistically significant, though the giving in the UG_AMB_ correlated more strongly with MAO (*r* = 0.44, *p* < .0001) or expectation (*r* = 0.31, *p* = .001) in the UG_STD_. On the other hand, elimination of those strong over-estimators of rejection hardly affected the correlation between the effect of intentionality manipulation on giving (i.e., giving in the UG_STD_ – UG_AMG_) and its effect on MAO (*r* = -0.06, *p* = .524) or expectation (*r* = 0.02, *p* = .860).  **Modulation of Giving by CRT**  Conceptually replicating the earlier studies with the DLPFC cortical thickness [18, 27, 28], the CRT score was positively correlated with the difference in giving between the DG and the UG_STD_ (*r* = .19, *p* = .042) (Fig. 1). Furthermore, the CRT score negatively correlated with giving only in the DG (*r* = -.29, *p* = .001), but not significantly with giving in the UG_STD_ (*r* = .18, *p* = .050) or the UG_AMB_ (*r* = .13, p = .152)) (Fig. 1B). When the participants were categorized into high CRT scorer who made no intuitive answer and low CRT scorers who made at least one intuitive answer (n = 65), the interaction effect of the categorized CRT and the three games in the repeated measure analysis of variance of giving was significant (*F*(2, 238) = 7.41, *p* = .001, *η*^2^ = .059).  We then focused our analysis on the comparison between the UG_STD_ and the UG_AMB_. The main effect in a repeated measure AVOVA of the proposer’s giving by the game type and the CRT, the main effect of the games was significant (*F*(1, 119) = 7.26, *p* = .008, *η*^2^ = .058). The main effect of the CRT was not significant (*F*(1, 119) = 0.88, *p* = .350, *η*^2^ = .007). The game type by CRT interaction effect was not significant, either (*F*(1, 119) = 0.09, *p* = .769, *η*^2^ = .001). The last two results indicate that the CRT did not modulate the effect of the intention manipulation on giving.  **Modulation of MAO and anticipation of rejection by CRT**  We next examined if the CRT differentially affected the levels of MAO in the three UG games. The interaction effect of the CRT and the three game types was significant (*F*(2, 238) = 10.22, *p* < .0001, *η^2^* = .079). As shown in Fig. 5A, the game differences in MAO were smaller in the high CRT scorers than in the low CRT scorers, especially the difference between the UG_STD_ and the other two games. The interaction effect of categorized CRT by the UG_STD_ versus the UG_AMB_ was also significant (*F*(1, 119) = 5.43, *p* = .021, *η^2^* = .044). The two-game difference was more pronounced in the low CRT scorers (*M for the difference* = 114.29 ± 37.68; *F*(1,55) = 36.96, *p* < .0001, *η^2^* = .402) than the high CRT scorers (*M* = 53.85 ± 25.94; *F*(1,64) = 17.19, *p* < .0001, *η^2^* = .212). That is, intentionality of the proposer had a larger effect on MAO among the low CRT scorers than the high CRT scorers, suggesting that MAO in the UG_STD_ is more strongly affected by intuitive drive for rejecting unfair offers among the former than the latter players. Comparable results were obtained regarding the anticipated rejection of unfair offers by other players (Fig. 5B); the two-game difference was more pronounced in the low CRT scorers (*M* = 16.43 ± 6.51; *F*(1,55) = 25.59, *p* < .0001, *η^2^* = .318) than the high CRT scorers (*M* = 12.31 ± 4.99; *F*(1,64) = 24.28, *p* < .001, *η^2^* = .275), although the interaction of the categorized CRT and the anticipated rejection was not statistically significant (*F*(1, 119) = 1.04, *p* = .310, *η^2^* = .009). |
| --- | --- | --- |
